# Hurricane air-sea drag saturation and sea-state dependence revealed by surface drones

**DOI:** 10.1126/sciadv.aec7422

**Published:** 2026-05-27

**Authors:** Gregory R. Foltz, Dongxiao Zhang, Lev B. Looney, Andrew M. Chiodi, Jun A. Zhang, Chidong Zhang, Edoardo Mazza, Nan-Hsun Chi, Edward D. Cokelet

**Affiliations:** ^1^ NOAA Atlantic Oceanographic and Meteorological Laboratory, Miami, FL, USA.; ^2^ University of Washington Cooperative Institute for Climate, Ocean, and Ecosystem Studies, Seattle, WA, USA.; ^3^ NOAA Pacific Marine Environmental Laboratory, Seattle, WA, USA.; ^4^ University of Miami Cooperative Institute for Marine and Atmospheric Studies, Miami, FL, USA.; ^5^ University of Miami Rosenstiel School of Marine, Atmospheric, and Earth Science, Miami, FL, USA.

## Abstract

The ocean supplies energy for tropical cyclones (TCs) and slows their winds through surface friction, which exerts a force on the ocean termed wind stress. The drag coefficient (*C*_d_) is the key parameter that converts wind speed to wind stress and is currently estimated in forecast models from incomplete data collected in low-to-moderate ocean winds. Here, we use measurements from 11 Atlantic hurricanes to quantify *C*_d_ in winds up to 44 meters per second and surface waves up to 14 meters. It is found that *C*_d_ levels off, as wind speed surpasses 30 meters per second but does not decrease appreciably as suggested by previous indirect methods. Interaction of the wind and wave fields causes *C*_d_ to be 20 ± 2% higher on the motion-left side of a storm, where wind and waves are misaligned, than on the right. These results quantify directly a fundamental TC air-sea interaction parameter and demonstrate the importance of distinct TC quadrant-specific wind-wave interactions.

## Teaser

The ocean’s frictional effect on hurricane winds saturates but does not decrease and is larger when wind and waves are misaligned.

## INTRODUCTION

Tropical cyclones (TCs) intensify primarily through the conversion of ocean heat energy into rotational energy of the TCs’ winds (*1*). In the process, wind momentum is transferred from the TC to the ocean, acting to slow the TC’s near-surface winds while generating ocean currents, surface waves, storm surge, and mixing that cools the ocean’s surface and acts as a negative feedback on TC intensity (*2*). It is critical for forecast models to simulate this momentum flux properly (*3*). However, models cannot resolve the small spatial and temporal scales explicitly, instead typically requiring parameterization in terms of the mean wind speed at a height of 10 m (*U*_10_) relative to surface ocean currents

$$|\tau |=\rho_{a}C_{d}U_{10}^{2}$$ (1)

where |τ| is the magnitude of the momentum flux (i.e., wind stress, τ), $\rho_{a}$ is the density of air, and *C*_d_ is an empirically determined drag coefficient. The presence of surface waves can modify the momentum flux (*4*–*7*); thus, wave-dependent parameterizations are important, especially as wave conditions can vary dynamically in TCs. However, due to extremely limited direct observations, wind stress direction and *C*_d_ in TCs and their relationships with surface waves are poorly known, hindering TC forecast model advancement (*6*, *7*). Observations in TCs are often unable to provide direct estimates of *C*_d_, which require resolving the small turbulence scales necessary to quantify τ

$$|\tau |=\rho_{a}\sqrt{{\left(\bar{u^{\prime }w^{\prime }}\right)}^{2}+{\left(\bar{v^{\prime }w^{\prime }}\right)}^{2}}$$ (2)

In contrast to the parameterization in Eq. 1, the covariance flux method in Eq. 2 enables direct calculation of τ. Here *u*′, *v*′, and *w*′ are deviations of the wind components from their mean values along the direction of the mean horizontal wind, across the direction of the mean horizontal wind, and vertically, respectively. The mean values are indicated by overbars and are relative to the measured surface ocean currents. The definition in Eq. 2 can alias negative values (i.e., upward momentum flux) into positive values, mainly when winds are weak. With high-quality measurements and when combined with Eq. 1, the direct covariance method is considered the highest standard for calculating *C*_d_ over the ocean. However, the technique is difficult to use, especially in strong winds, because of the need for continuous high-frequency (20 Hz) measurements of three-dimensional wind velocity that are uncontaminated by motion of the measurement platform and not affected by sea spray. These measurements have traditionally been carried out using moored buoys or research vessels. Additional difficulties arise when attempting to estimate *C*_d_ in strong winds because of their infrequent occurrence and the formidable challenges of measuring near the surface of the ocean at high resolution under extreme conditions. As a result, most previous observational estimates of *C*_d_ in strong winds either use measurements over the ocean and apply indirect methods that make assumptions about the vertical structure of the mean winds or ocean currents (*8*–*10*) or are from laboratory experiments (*11*, *12*). Direct measurements of τ in the ocean have been limited to *U*_10_ less than 27 m s^−1^ and without inclusion of concurrent surface wave observations (*13*, *14*).

**Fig. 1.**
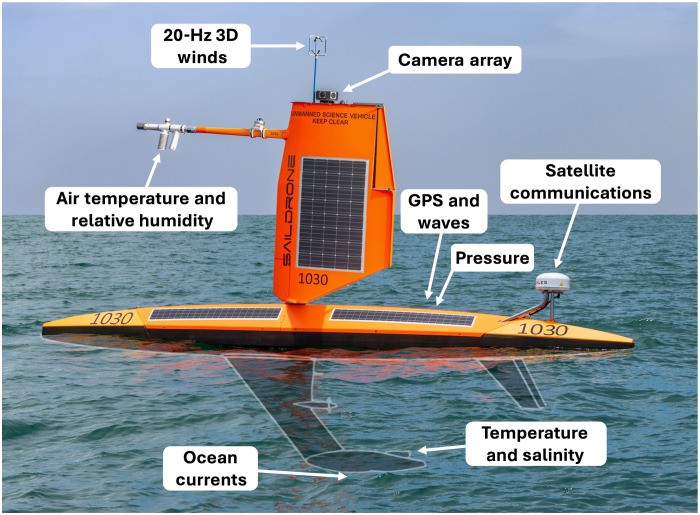
Saildrone Explorer USV for TC observations. Instruments used for the calculation of *C*_d_ and its dependence on sea state are indicated.

During the past decade, uncrewed surface vehicles (USVs) have been used increasingly to collect high-quality observations near the air-sea interface across a wide range of weather conditions (*15*). Key advantages of USVs compared to fixed platforms and ships are that USVs can be piloted remotely and directed into extreme conditions without putting people at risk. The USVs used in this study (Fig. 1) normally travel at about 0.5 to 1.5 m s^−1^, depending on near-surface winds and currents. Therefore, advanced planning and strategic vehicle piloting, based on evolving hurricane track and intensity forecasts and ocean-atmosphere conditions, are required to maximize the chances of acquiring data in the core of a hurricane. This enabled the collection of a large number of USV observations in TCs during the 2021–2024 Atlantic hurricane seasons, including three-dimensional 20-Hz winds and surface wave properties (www.pmel.noaa.gov/usv-hurricane/). Here, using Eqs. 1 and 2 with USV measurements, we directly quantify the air-sea momentum flux and the dependence of *C*_d_ on wind speed and wave properties in 20-min–averaged *U*_10_ up to 44 m s^−1^ and significant wave heights as large as 14 m. Hereafter, except where otherwise noted, all wind speeds reported have been adjusted to a height of 10 m after motion correction, as described in Materials and Methods.

## RESULTS

### Ocean-atmosphere observations under extreme conditions

**Fig. 2.**
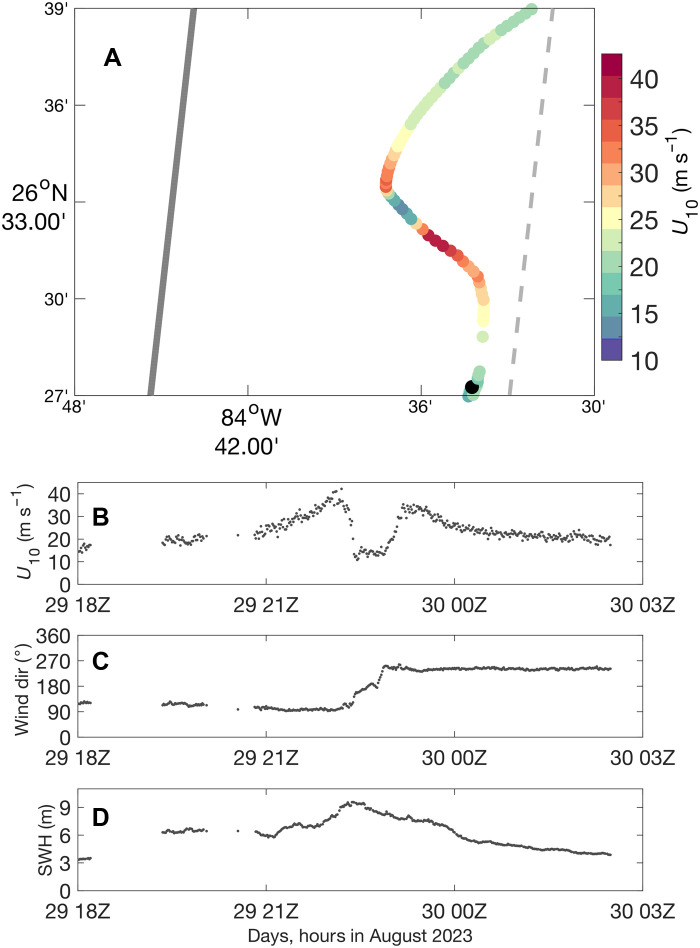
Data collected by a saildrone in Hurricane Idalia. (**A**) Path of the saildrone (dots colored on the basis of measured *U*_10_), track of Hurricane Idalia as it moved northward (solid gray line), and eastward extent of the radius of maximum wind (dashed gray line). Five-minute–averaged (**B**) *U*_10_, (**C**) wind direction, and (**D**) significant wave height measured by the saildrone. dir, direction. SWH, significant wave height.

**Fig. 3.**
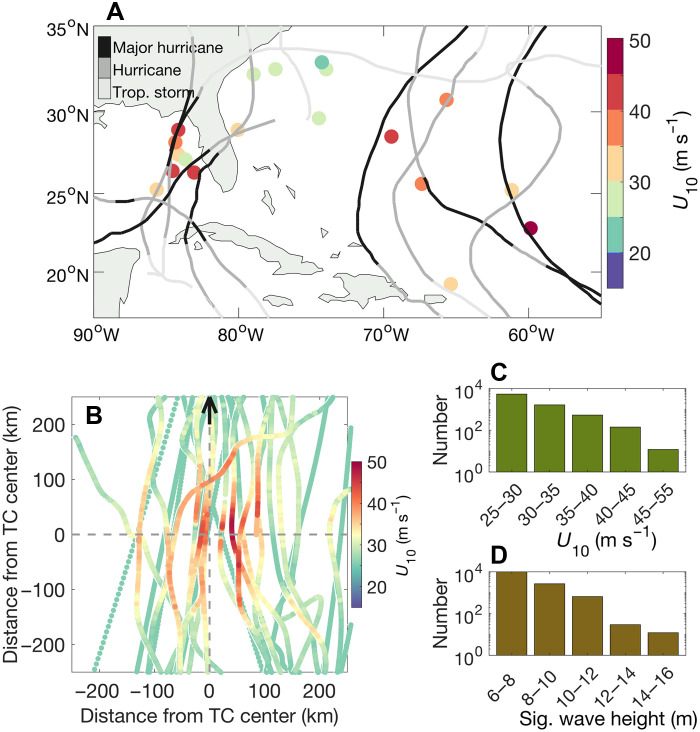
TC data coverage. (**A**) Locations of saildrone TC intercepts used in this study (dots). Color indicates the maximum measured 1-min–averaged *U*_10_. Gray lines show the TC tracks shaded by intensity: tropical storm (18 to 32 m s^−1^), hurricane (33 to 49 m s^−1^), major hurricane (50+ m s^−1^). Trop., tropical. (**B**) Colors: *U*_10_ measured along the paths of the saildrones with TC intercept points shown in (A). Storm-following coordinates are used: The *x* axis represents the distance to the right of each TC’s center, and *y* axis is the distance ahead of each TC’s center along its track. The black arrow indicates the direction of the TCs’ motion. (**C**) Histogram of 1-min *U*_10_ from all TC intercepts shown in (A). (**D**) Histogram of 1-min significant wave height measured in all TCs. Note the logarithmic scales in (C) and (D). Sig., significant.

**Fig. 4.**
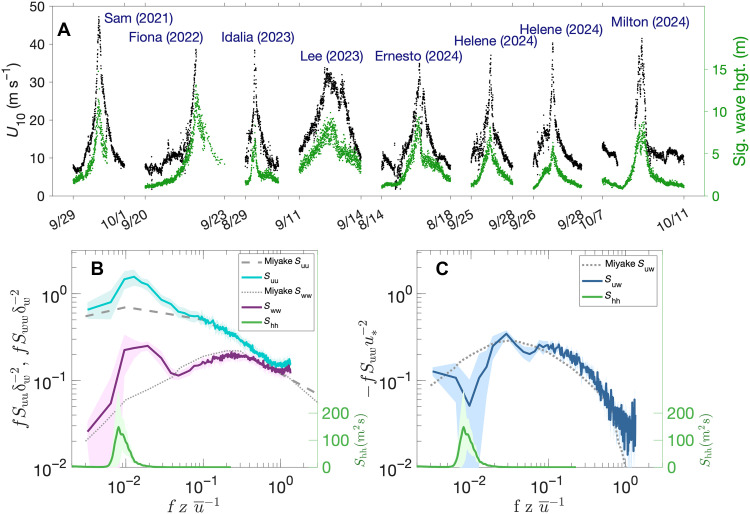
Saildrone wind and wave data in TCs. (**A**) Five-minute–averaged horizontal wind speed (black) and significant wave height (green) from eight hurricane intercepts with the strongest measured winds. Dates on the *x* axis denote the start and end of each individual time series, except only the start month and day are given for Hurricane Idalia because of its short duration. hgt., height. (**B**) Normalized power spectra of 20-Hz horizontal wind speed (blue), vertical wind speed (purple), and 4-Hz wave height (green), all for *U*_10_ > 25 m s^−1^. (**C**) Same as (B) but for the horizontal-vertical wind cross-spectrum (blue). Colored shading in (B) and (C) shows one SD across all 20-min segments used to calculate the mean spectra. In (B), gray curves show the corresponding universal horizontal (dashed) and vertical (dotted) wind spectra from (*18*). The gray dotted curve in (C) is the wind cross-spectrum from (*18*).

The data were acquired by specialized remotely operated Saildrone USVs (hereafter “saildrones”; Fig. 1) that were directed into the paths of Atlantic hurricanes (TCs with maximum sustained wind speed > 33 m s^−1^) during 2021 to 2024 (*16*). Figure 2 shows an example of the track of one of the saildrones and its wind and wave measurements as it traveled close to the center of Hurricane Idalia in August 2023. In total, 42 saildrone intercepts of TCs resulted in measured 1-min–averaged tropical storm–force wind speeds (*U*_10_ > 18 m s^−1^). Here, an intercept is defined as a saildrone obtaining data within 300 km from the center of a tropical storm or hurricane. We use 19 of the intercepts with the longest records of strong winds (Figs. 3 and 4), nine of which contain 1-min–averaged hurricane-force *U*_10_. The longest periods of time with continuously measured tropical storm– and hurricane-force *U*_10_ in a single TC are 43 and 4.3 hours, respectively, in hurricanes Lee and Sam. The saildrones were preferentially positioned to the right of the forecasted TC tracks (e.g., Fig. 2), with the goal of measuring the strongest winds in the storm motion–relative front-right quadrant. However, because of imperfect TC track forecasts and the slow movement of the saildrones, they ended up sampling all four storm motion–relative quadrants throughout the western Atlantic and west of Florida (hereafter “Gulf”): 33% of the data in winds (18+ m s^−1^) are from the front-right quadrant, 24% from the rear-right quadrant, 23% from the rear-left quadrant, and 19% from the front-left quadrant.

In the strongest hurricanes sampled, wind speed and wave height increased abruptly as the storm center approached the saildrones (Figs. 3B and 4A). Maximum significant wave heights range from 6 to 15 m, with the highest values observed in category-4 hurricanes Sam (2021) and Fiona (2022) (table S1). For both, there is a nearly symmetric peak in wave height because the saildrones traveled through the front-right and then rear-right quadrants, where the wind direction and wave propagation tend to be most closely aligned (*5*, *17*). In contrast, in Lee (2023) and Ernesto (2024), there are abrupt decreases in wave height after their peaks. In those hurricanes, the saildrones traversed the weaker rear-left quadrants, where generally the wind fetch is shorter and swells propagate outward from the storm’s center as winds rotate across or against them. The saildrones sampled a wide variety of storm sizes, translation speeds, and intensities. The radius of sustained hurricane-force wind speed ranged from 35 km in Idalia (2023) to 205 km in Lee (2023). The translation speed varied from 3 m s^−1^ in Hurricane Lee to 8 m s^−1^ in Milton (2024). The maximum 1-min–averaged *U*_10_ was 48 m s^−1^ in Hurricane Sam, when it was category 4 with estimated maximum sustained wind speed of 65 m s^−1^.

Although this is the first time that continuous high-frequency time series of hurricane-force winds have been measured near the air-sea interface, the power spectra of TC horizontal and vertical winds show the expected relationships with frequency observed in weaker winds (*18*, *19*) when normalized for measurement height and mean wind speed (Fig. 4B). Wind energy decreases with increasing normalized frequency in the inertial subrange (normalized frequencies >0.2 for vertical wind and >0.01 for horizontal wind), where there is a cascade of energy to smaller temporal and spatial scales. At lower normalized frequencies outside the inertial subrange, where turbulent energy is generated in larger eddies, the energy increases with increasing normalized frequency. The wind spectra have peaks near the dominant surface wave frequency, and the peak is most pronounced in the vertical wind component, suggesting a close association of TC winds with energetic ocean waves and a consistency with wave-induced modulations of the near-surface flow (*20*–*22*). There is a decrease in the horizontal-vertical wind cospectrum at the dominant wave frequency (Fig. 4C), which was also found in a previous analysis of direct near-surface wind measurements in a TC (*14*).

### *C*_d_ and its dependence on wind speed and sea state

The saildrones measured all variables needed to quantify *C*_d_ and its dependence on surface wave properties, including three-dimensional wind velocity sampled at 20 Hz and at a height of 3.4 m and sea surface elevation recorded at 4 Hz (see Materials and Methods). From the high-frequency winds, |τ| was calculated from Eq. 2 using 20-min means and the 20-Hz deviations from them, and then *C*_d_ was calculated from Eq. 1.

**Fig. 5.**
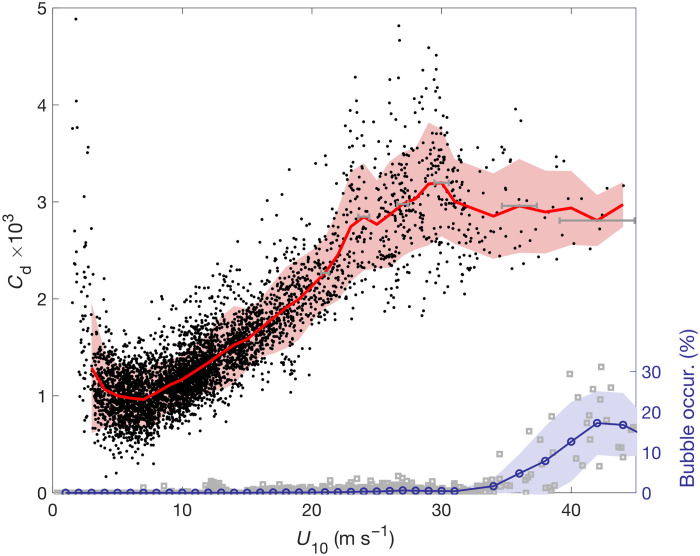
*C*_d_ as a function of wind speed. Black dots represent *C*_d_ values calculated over continuous 20-min segments. Red line shows averages in each bin (2 m s^−1^) with overlap (1 m s^−1^) for *U*_10_ < 30 m s^−1^ and in bins (2 m s^−1^) with overlap (2 m s^−1^) for *U*_10_ > 30 m s^−1^. Red shading is one SD from the mean. Horizontal gray lines indicate *U*_10_ uncertainty based on the bin averages of 20-min mean SDs of 20-Hz *U*_10_ (see Materials and Methods). Gray squares are air bubble occurrence in each 20-min segment, calculated as the percentage of 1-Hz measurements with air bubbles detected. The blue line with circles shows the binned values using the same averaging as for *C*_d_ (red line), and blue shading is one SD from the mean. occur., occurrence.

At low to moderate wind speeds (<20 m s^−1^), *C*_d_ calculated from the saildrone data shows a robust increase with increasing wind speed (Fig. 5) that agrees well with previous direct covariance estimates that used only the along-wind *C*_d_ (*23*). The difference in definition leads to about a 5 to 10% difference between the total *C*_d_ and along-wind *C*_d_. Across all TCs observed by the saildrones, there are 790 distinct 20-min calculations of *C*_d_ for *U*_10_ > 18 m s^−1^ (i.e., tropical storm–force wind) and 49 for *U*_10_ > 33 m s^−1^ (i.e., hurricane-force wind). There is a slowing of the increase in *C*_d_ at about 25 to 30 m s^−1^, followed by a leveling off and very little change in *C*_d_ as *U*_10_ increases to 44 m s^−1^. The along-wind component dominates *C*_d_ for most wind speeds, although the cross-wind component becomes more important as *U*_10_ increases above 25 m s^−1^ (fig. S1), consistent with previous results that show increasing misalignment of wind and wind stress in high winds (*10*). The peak in cross-wind *C*_d_ for *U*_10_ = 25 to 30 m s^−1^ (fig. S1B) is consistent with the peak in *C*_d_ in the same *U*_10_ range (Fig. 5).

We leverage near-surface seawater conductivity measurements in the conductivity-temperature-depth (CTD) sensor to provide context for the saturation behavior of *C*_d_ in winds greater than 30 m s^−1^. Under these extreme conditions, breaking waves and streaks of foam interrupt the continuous flow of seawater past the conductivity cell, resulting in anomalously low conductivity values. We interpret these values as a proxy for bubble concentration (see Materials and Methods). It is found that air bubbles are infrequent below wind speeds of about 32 m s^−1^ (Fig. 5). This is followed by a steady increase in their occurrence, as wind speed increases to about 42 m s^−1^ and *C*_d_ changes very little. Taking the frequency of air bubbles as a proxy for wave breaking and foam (*24*, *25*), the results suggest that their increased occurrence helps to limit wave steepness and surface roughness, preventing *C*_d_ from increasing (*26*). Larger and more frequent wave breaking also generates more sea spray, which decreases the downward flux of momentum and *C*_d_ (*27*).

The variance of the individual *C*_d_ values (black dots in Fig. 5) also increases with *U*_10_ from 20 m s^−1^ up to about 30 m s^−1^. One interesting feature is that the peak in variance in this wind range corresponds to the maximum in *C*_d_. The variance then decreases as *U*_10_ increases further. To test the significance of the difference in SDs, we randomly subsampled *C*_d_ in the range of 25 to 30 m s^−1^, where the *C*_d_ variance is highest, to match the number of *C*_d_ observations in the range of 30 to 40 m s^−1^ (i.e., 131) and repeated 1000 times. We found that the number of *C*_d_ observations in the range of 30 to 40 m s^−1^ sufficiently samples the normal distribution with the given SD: The 95% confidence interval is 5.0 to 6.3 × 10^−4^ for the subsampled *C*_d_ SD (*U*_10_ = 25 to 30 m s^−1^, with mean SD of 5.7 × 10^−4^) and does not overlap with the SD of 4.3 × 10^−4^ for *C*_d_ when *U*_10_ is between 30 and 40 m s^−1^. Results are similar if the calculations are limited only to data from TCs with *U*_10_ exceeding 37 m s^−1^, indicating that the decrease in the number of TCs sampled cannot explain the decrease in variance as *U*_10_ increases above 30 m s^−1^ (fig. S2A).

### Impact of wind-wave misalignment on *C*_d_

**Fig. 6.**
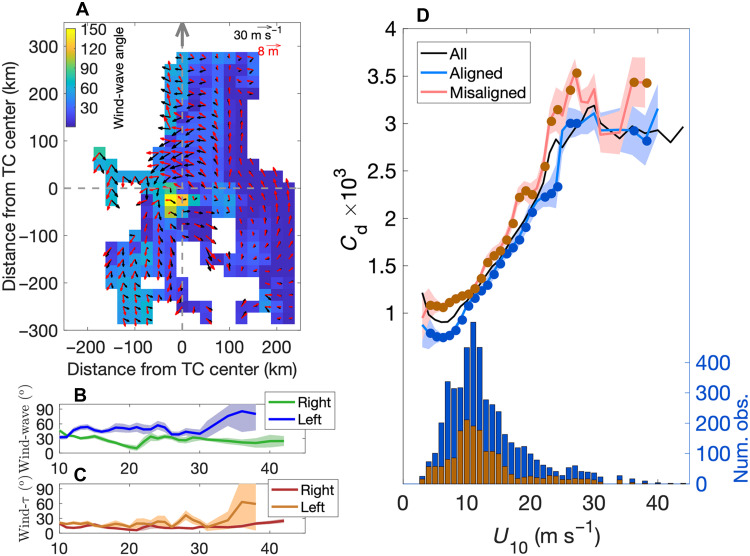
Wind-wave misalignment and its impact on *C*_d_. (**A**) Magnitude of wind-wave angle in storm-following coordinates, averaged over all storms (shading). The gray arrow at the top shows the direction of storm motion. Black and red arrows show mean wind direction and wave direction, respectively. The arrow lengths indicate wind speed (*U*_10_) and significant wave height, with scales shown in the upper right. Dashed gray lines separate the TC quadrants. (**B**) Wind-wave angle as a function of wind speed, averaged on the motion-right sides of the TCs (green) and motion-left sides (blue). (**C**) Angle between wind vector and wind stress (τ) averaged on the motion right (red) and motion left (orange). Shading shows two SEs. Data are plotted only for *U*_10_ > 10 m s^−1^, when storm quadrants are most meaningful. (**D**) *C*_d_ using all data as in Fig. 3 (black), using only data with misaligned wind and waves (>60°; red) and aligned wind and waves (<20°; blue). Values with brown and blue dots are significantly different from each other at the 5% level based on a two-sample Student’s *t* test. Shading shows two SEs. Histograms show the number of 20-min segments with misaligned (brown) and aligned (blue) wind and waves. Num. obs., number of observations.

The combination of direct wind stress and wave measurements from the saildrones provides valuable insight to the effects of wind-wave misalignment on *C*_d_ and their dependence on TC quadrant. The saildrones’ data distribution is weighted more heavily to the motion-right sides of the TCs, but all quadrants were well sampled. For *U*_10_ > 18 m s^−1^, there are 280 continuous 20-min data segments in the front-right quadrant, 209 in the rear-right quadrant, 205 in the rear-left quadrant, and 166 in the front-left quadrant. There is also a reasonable distribution of data across different TCs for most wind speeds (fig. S2A). In the strongest winds within ~50 km from a hurricane’s center, wind and waves tend to be most closely aligned in the front-right quadrant and adjacent portions of the rear-right and front-left quadrants (Fig. 6A) (*5*, *17*, *28*, *29*). In those areas, the rotating wind combines with the hurricane’s translation to generate waves that propagate toward the front and front-left of the storm, largely aligned with the cyclonic wind. However, in the rear-left quadrant, waves continue to propagate toward the left or front left of the storm while the rotating wind crosses or opposes them, generating wind-wave misalignment angles ($\theta_{\text{ww}}$) that can approach 180°. Here, $\theta_{\text{ww}}$ is defined as the angle between the swell (6- to 20-s wave period) propagation direction and wind direction. Differences in $\theta_{\text{ww}}$ within TCs have been shown to generate changes in sea surface roughness and *C*_d_ (*5*, *29*), although there is not a consensus on whether larger $\theta_{\text{ww}}$ results in larger or smaller *C*_d_ (*5*, *30*). It is also unclear how wind-wave misalignment might affect the transfer of momentum at the ocean’s surface (i.e., τ) because there are no previous direct measurements of τ and $\theta_{\text{ww}}$ in *U*_10_ exceeding 27 m s^−1^.

The storm-relative asymmetry in wind-wave alignment can be seen in Fig. 6B, which shows that for wind speeds greater than 10 m s^−1^, the average $\theta_{\text{ww}}$ is always larger on the left side of the TC than the right. When the wind and waves are nearly aligned, the wind and τ are also well aligned because the wind-induced turbulence is oriented mostly in the direction of the wind and waves (*31*). As a result, the angle between the wind and τ ($\theta_{\mathrm{w\tau}}$) is small on average on the right side of a TC (Fig. 6C). On the left side, where $\theta_{\text{ww}}$ is larger, the wind stress is less well aligned with the wind and more aligned with the waves. This results in larger positive values of $\theta_{\mathrm{w\tau}}$ for most wind speeds, meaning that τ is oriented to the right of the downwind direction. This is consistent with the observed wind-wave orientations in most TC quadrants (Fig. 6A) and the sign of the correlation between $\theta_{\text{ww}}$ and the cross-wind component of τ (i.e., the cross-wind τ is directed to the right of the mean downwind direction).

To quantify the impact of $\theta_{\text{ww}}$ on *C*_d_, we calculate the mean *C*_d_ for cases with large misalignment ($\theta_{\text{ww}}$ > 60°) (*30*) and with nearly aligned wind and waves ($\theta_{\text{ww}}$ < 20°). For most wind speeds, *C*_d_ is larger for greater wind-wave misalignment (Fig. 6D). As a result, for wind speeds > 18 m s^−1^, *C*_d_ is on average 20 ± 2% higher on the motion-left side of TCs than on the right and 23 ± 2% higher in the rear-left quadrant compared to the rear-right quadrant. Here, the uncertainties are calculated as two SEs. Results are similar when only the along-wind component of *C*_d_ is used, although the cross-wind *C*_d_ is also significantly higher for misaligned wind and waves in weak-moderate winds (fig. S3). The largest differences in *C*_d_ between misaligned and aligned wind and wave cases occur for wind speeds of 22 to 28 m s^−1^ (Fig. 6D), providing another possible explanation for the larger variance of *C*_d_ in that wind speed range (Fig. 5). The largest cross-wind component of *C*_d_ occurs in that wind speed range (fig. S1B), consistent with the misaligned wind and wind stress (Fig. 6C).

Notably, results are insensitive to the exact thresholds used to define aligned and misaligned (fig. S4A). We also found that the variability of wind direction and wave direction is only weakly dependent on wind speed: The SD of 20-Hz wind direction, calculated in each 20-min segment, increases from 7° for *U*_10_ = 0 to 10 m s^−1^ to 8° for *U*_10_ > 40 m s^−1^, while the SD of 2-min wave direction decreases from 13° to 11° for the same change in *U*_10_ (fig. S5, C and D). As a result, the larger uncertainty in wind-wave alignment angle at high winds results mainly from the reduction in the number of measurements. The larger values of *C*_d_ for more misaligned wind and waves possibly result from greater surface roughness, as wind at an angle to the waves has a smaller component along the wave propagation direction, and, for $\theta_{\text{ww}}$ > 90°, a component against the waves (*30*, *32*).

## DISCUSSION

Our results provide the first direct quantification of the air-sea momentum flux and *C*_d_ in hurricane-force winds using field observations and have important implications for understanding and modeling air-sea interactions in TCs. We found that for *U*_10_ in the range of 25 to 42 m s^−1^, *C*_d_ saturates at a value of about 2.5 to 3 × 10^−3^ and does not decrease appreciably as many previous studies using indirect methods have indicated (fig. S6). Our *C*_d_ estimates for *U*_10_ > 25 m s^−1^ are larger than those currently used in the operational hurricane prediction models Hurricane Weather Research Forecast (HWRF) (*33*) and Hurricane Analysis and Forecast System A (HAFS-A) (fig. S7) (*34*). Those widely used models use a *C*_d_ that peaks and then decreases for *U*_10_ > 30 m s^−1^, based on previously reported values (fig. S6), with possible implications for TC intensity biases in HWRF and HAFS-A. The differences between our results and previous results are likely due at least, in part, to data limitations, with previous studies sometimes analyzing only one TC. The assumptions and simplifications required to infer *C*_d_ indirectly from vertical wind profiles (*35*) and the ocean momentum budget (*9*, *10*) also likely contribute to the large spread found between previous results (fig. S6). Collocated indirect and direct measurements of the momentum flux and its height dependence would be extremely valuable for testing this hypothesis and the validity of a constant flux layer near the surface (*36*, *37*).

**Fig. 7.**
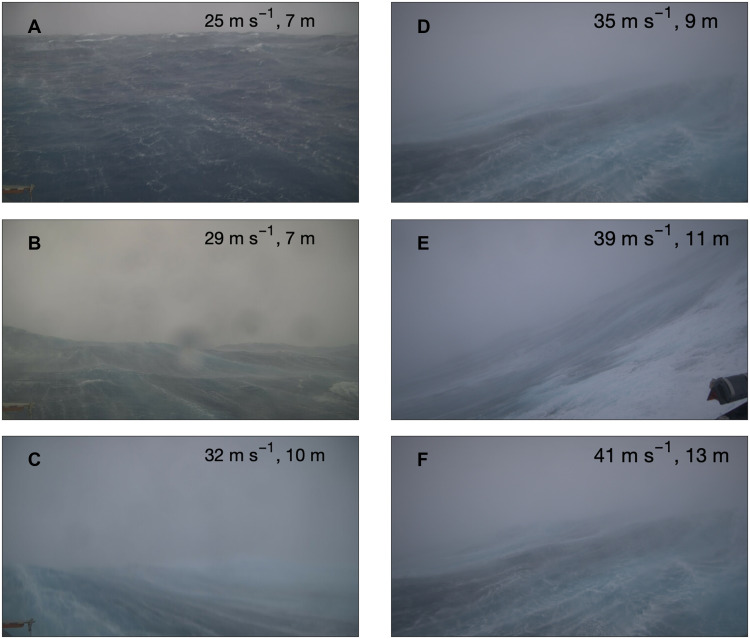
Near-surface conditions in varying wind and waves. Images were taken by Saildrone Explorer USVs. (**A** to **C**) Hurricane Lee on 12 and 13 September 2023 at (A) 07:40, (B) 09:40, and (C) 17:40 local time (12:10, 14:10, and 22:10 UTC, respectively). (**D**) Hurricane Milton on 26 September 2024 at 17:10 local time (22:40 UTC). (**E** and **F**) Hurricane Sam on 29 September 2021 at (E) 13:15 local time (17:15 UTC) and (F) 12:00 local time (16:00 UTC). The 20-min–averaged *U*_10_ and significant wave height, centered on the time of the image, are indicated.

Previous studies have provided possible explanations for the saturation of *C*_d_ in high winds and the large scatter of *C*_d_ for different surface wave conditions (*4*, *5*, *11*). Visual images from the saildrones also provide insight to the possible causes of the sharp increase in *C*_d_ for wind speeds of 20 to 30 m s^−1^, the peak in its variance at 25 to 30 m s^−1^, and the saturation of *C*_d_ above 30 m s^−1^ (Fig. 7). The state of the ocean’s surface changes markedly as *U*_10_ increases from 25 to 32 m s^−1^ (Fig. 7, A to C), with foam much more prevalent in the image (32 m s^−1^) . In contrast, between 32 and 41 m s^−1^, visually the ocean’s surface does not appear to change as much. The foam characteristics and distributions from these images are consistent with the saturation of *C*_d_ for *U*_10_ above about 30 m s^−1^: Likely the smoother ocean surface due to foam helps put a cap on ocean surface roughness and *C*_d_. Other processes related to foam generation, such as wave breaking and flow separation as the wind blowing over wave crests loses contact with the surface, have also been hypothesized to limit *C*_d_ in strong winds (*11*). The less variable ocean surface conditions above 30 m s^−1^ seen in Fig. 7 are consistent with the reduced variance of *C*_d_ in the strongest winds compared to the range of 25 to 30 m s^−1^.

To quantify more objectively changes in the ocean’s surface with changing wind speed, we analyzed 97 visual images recorded by saildrones in seven different hurricanes. In each image, the ocean pixels were identified, and the average brightness of the ocean’s surface across all pixels was determined, with values ranging from 0 (i.e., black) to 1 (white) (see Materials and Methods and fig. S8). The results show that as *U*_10_ increases above about 30 m s^−1^, the ocean’s surface becomes brighter on average, indicating an increasing prevalence of air bubbles and foam (fig. S9). This occurs despite generally darkening skies with increasing wind speed in hurricanes, which would tend to reduce ocean brightness. There appears to be an abrupt transition around *U*_10_ = 30 m s^−1^, with stronger winds associated with fewer cases of low brightness. The mean brightness for *U*_10_ > 30 m s^−1^ is 0.48 compared to 0.45 for *U*_10_ < 30 m s^−1^, and the difference is statistically significant at the 5% level based on a two-sample *t* test. The SD of ocean brightness is also slightly higher for *U*_10_ < 30 m s^−1^ (0.07) compared to *U*_10_ > 30 m s^−1^ (0.06), consistent with the larger variance inferred from Fig. 7 and seen in *C*_d_ in Fig. 5.

The measurements from the saildrones in hurricanes are often below the wave crests. Therefore, it is possible that our *C*_d_ estimates are a lower bound on the true *C*_d_ because our calculation did not include the near-surface wave-induced pressure flux p∂h/∂x, where *p* is near-surface pressure, *h* is sea surface elevation, and *x* is in the direction of wave propagation (*38*, *39*). Calculation of the pressure flux near the ocean’s surface is extremely difficult because of the requirement of highly accurate pressure readings that are not contaminated by dynamic pressure effects. In a controlled laboratory wave tank, the pressure flux was measured at a height of about 2 cm above the surface in *U*_10_ of 8 to 27 m s^−1^ and wave steepness (amplitude times wave number) of 0.032 to 0.038 (*35*), which is close to the steepness we observed from saildrones in hurricanes (~0.01 to 0.03). Averaged across all five experiments, the pressure flux–induced *C*_d_ was 0.2 × 10^−3^. It is therefore possible that our *C*_d_ estimates are biased low by a similar amount, especially for high winds. Notably, the wave tank measurements have their own limitations compared to open-ocean observations in that the wave tank boundaries may affect the *C*_d_ pressure flux and waves. We also note that model experiments suggest an even greater contribution from the pressure flux (*39*).

As winds increase from 20 to 30 m s^−1^, we showed that the combination of a rapidly changing sea state and marked spatial variations of wind-wave alignment leads to greater deviations of *C*_d_ from a strict dependence on wind speed. Numerical models are likely to see improvements in simulating TCs if they incorporate wind stress parameterizations that account for larger *C*_d_ on TCs’ motion-left sides, and especially the rear-left quadrant, where wind-wave misalignment is consistently greater. However, at a more fundamental level, wind-wave coupling holds the greatest promise for improving simulations of the air-sea momentum flux in strong winds. In addition, we found greater misalignment of the wind stress and wind vector on TCs’ motion-left sides, likely due, in part, to the greater wind-wave misalignment. Failure to account for these spatial variations in wind stress in forecast models could potentially lead to errors in wind-driven upwelling, ocean surface cooling, and TC intensity. Additional targeted near-surface ocean observations in hurricanes and further development of in situ platforms capable of direct measurements of wind stress, directional wave spectra, sea spray, and foam are needed to further advance our understanding of these complex air-sea processes in TCs. Ideally, the advanced understanding will improve parameterizations of *C*_d_ that account for not only wind speed but also sea state, including wave height and direction and amount of foam and sea spray.

## MATERIALS AND METHODS

### Saildrone data

The primary dataset consists of three-dimensional wind velocity, air temperature, relative humidity, sea surface temperature (SST), sea surface salinity (SSS), wave height and period, surface pressure, ocean currents, and vehicle latitude, longitude, and acceleration measured by specialized short-wing Saildrone Explorer USVs (hereafter saildrones; Fig. 1) (*16*). We also use visual images acquired by the cameras mounted on the saildrones’ wings. Data from 19 distinct TC intercepts are used (see table S1). Wind is measured at a height of 3.4 m with a Gill WindMaster ultrasonic anemometer, air temperature and relative humidity at a height of 2.3 m with a Rotronic HC2-S3 probe, SST and SSS by a Seabird SBE 37 CTD mounted at a depth of 1.6 m, and ocean currents by a 300-kHz RDI Workhorse acoustic Doppler current profiler (ADCP). The anemometer also estimates air temperature from the travel times of ultrasonic pulses between its probes. Latitude, longitude, vehicle velocity, heading, and heave, as well as pitch, roll, yaw, and 3-axis acceleration and angular rate, are measured with a VectorNav-300 dual GPS-aided inertial navigation system (GPS/INS). The motion correction of the measured winds and their transformation into the Earth coordinate system follow (*40*), except that no additional postprocessing filtering is applied to the INS data. The GPS-aided INS on the saildrones includes an onboard microprocessor that performs internal filtering, including Kalman filtering to optimally couple GPS measurements with inertial sensor data. In addition, a vector processing engine uses a suite of algorithms that adaptively tune the Kalman filters in real time based on measurement uncertainties. The GPS/INS output is synchronized to the three-dimensional wind measurements at 20 Hz and is used directly for motion correction (*41*, *42*). It has been shown that the saildrone wind motion correction works as intended, removing the contributions from vehicle horizontal, vertical, and angular motion, including pitch, roll, and yaw.

The sampling frequencies are 20 Hz for wind velocity, GPS/INS, latitude, and longitude, 4 Hz for heave, and 1 Hz for air temperature, relative humidity, pressure, SST, SSS, and ocean currents. In strong winds and large waves, the 1-min–averaged wind measurement height has been as low as 2.2 m and the 20-Hz measurement height as low as −1.6 m (i.e., below the water line when the vehicle rolls more than 90°). However, all data with measurement heights less than 2 m are removed through quality control (QC) (see the “Saildrone data QC” section). More details of the saildrone vehicle and specifics of its sensors are provided in (*16*).

### Saildrone data QC

The Saildrone Explorer with full-height wing has been used extensively for research-quality ocean-atmosphere data collection in low-moderate winds over the past decade (*15*, *43*). The specialized short-wing saildrone was first used in 2021 for the purpose of collecting data in hurricanes (*44*). The anemometer is mounted atop the saildrone’s solid and rigid wing, which is used for wind propulsion and therefore changes direction relative to the wind. We performed QC on the saildrone wind measurements mainly to remove data likely affected by flow distortion from the saildrone’s wing, especially for large wind-wing and vehicle roll angles. Because of the specific wing design for propulsion, the vehicle sails more efficiently with its wing pointing into the wind; therefore, flow distortion is minimized for optimum wind measurements under most sailing conditions (*41*). Nevertheless, we remove all 20-Hz wind data when any of the following conditions are met: (i) The magnitude of the 1-min–averaged wind-wing angle exceeds 25°, (ii) the magnitude of the 20-Hz wind-wing angle exceeds 40°, (iii) the magnitude of the 1-min–averaged vehicle roll or pitch angle exceeds 20°, (iv) the magnitude of the 20-Hz roll or pitch angle exceeds 40°, or (v) the 1-min–averaged vertical wind magnitude exceeds 1 m s^−1^. The wind-wing angle criteria are similar to, although more restrictive than, the method typically used for direct covariance measurements from ship masts, which eliminates data when the wind-bow angle is greater than ~120° (i.e., >60° to either side of the bow-stern line) (*40*). Results for *C*_d_ are not very sensitive to the exact wind-wing, roll and pitch angles, and mean vertical wind QC thresholds (fig. S10).

In addition, we use data only with an anemometer error code of 00, indicating that all components are functioning properly. On the basis of visual inspection of the 20-Hz wind time series, we also remove segments of data that exhibit obvious symptoms of flow distortion: sudden reduction in mean horizontal wind with an anomalous increase in mean upward wind component and horizontal-vertical wind covariance (i.e., $u_{*}^{2}$).

### TC data

Three-hourly TC tracks and maximum 1-min–averaged TC wind speeds at a height of 10 m were obtained from the International Best Track Archive for Climate Stewardship (IBTrACS) database (*45*, *46*). Although the data are 3 hourly, the observations are mostly 6 hourly, with linear interpolation performed between them. The data are used to show TC tracks and intensities in Figs. 2 and 3 and to calculate the saildrones’ locations relative to TCs’ centers following (*47*), interpolating linearly to the locations of the saildrones (see also the “Wave direction” section below).

### Drag coefficient calculation

The drag coefficient (*C*_d_) is calculated as $u_{*}^{2}U_{10}^{-2}$, where $u_{*}^{2}=\sqrt{{\left(\bar{u^{\prime }w^{\prime }}\right)}^{2}+{\left(\bar{v^{\prime }w^{\prime }}\right)}^{2}}$. Here, *U_10_* is wind speed at a height of 10 m under neutral conditions and relative to the surface ocean velocity (see details later in this section); *u*′, *v*′, and *w*′ are deviations from each 20-min mean using 50% overlap with the previous 20-min mean and the overbar represents the mean calculated over the same 20-min period. We only use segments with at least 40% of the 20-Hz data available after QC, and the valid data are concatenated after removal of the questionable data. Overall, for measured wind speeds greater than 20 m s^−1^, 44% of the 20-Hz data are removed during QC. Although results are not very sensitive to the data availability threshold (fig. S10), the threshold is lower than what has been used in some previous studies (*48*). Therefore, we performed a more in-depth analysis of the impact of missing 20-Hz data on our estimates of *C*_d_. First, using the quality-controlled 20-Hz data from the saildrones, we found all 20-min segments with less than 10% missing data. We then found the 20-min segment of 20-Hz data with between 40 and 60% missing data that were closest in time to each segment with less than 10% missing data. We used the time positions of the missing data in the 40 to 60% missing data segment to mask out the data in the <10% missing data segment with the corresponding times relative to the start of the segment. We then calculated the mean wind speed and *C*_d_ from the original 20-min segment with <10% missing data and from the same segment with additional data removed.

Results show that there are differences in *C*_d_ of up to 2 × 10^−3^ for individual 20-min segments but very little mean bias (see fig. S11). The subsampled *C*_d_ with 40 to 60% data missing is 0.01 × 10^−3^ higher on average across all wind speeds and 0.02 × 10^−3^ lower on average for *U*_10_ > 20 m s^−1^. The SD is slightly higher for subsampled *C*_d_ across all wind speeds (0.66 × 10^−3^ compared to 0.64 × 10^−3^ for original data). On the basis of these results, we have confidence that our use of segments with up to 60% missing data does not significantly bias the *C*_d_ results at any wind speed and has an insignificant impact on *C*_d_ variance. We also found a high degree of stationarity in the 20-Hz wind speed and direction (fig. S5, B and C).

Before calculating *C*_d_, near-surface currents are subtracted from the measured 20-Hz wind velocity using data from the uppermost saildrone ADCP bin, which is at a depth of ~6 m. To adjust the mean measured wind to a height of 10 m (20-Hz data used to calculate *C*_d_ are not adjusted), we assume a logarithmic wind profile

$$U_{10*}=u_{z}+\frac{u_{*}\text{ln}\left(\frac{10}{z}\right)}{\kappa }$$

Here, *U*_10*_ is the wind adjusted to 10 m without adjustment for stability effects, *u_z_* is the wind speed measured at height *z*, u_*_ is friction velocity, and κ is the von Kármán constant (0.4). The neutral stability 10-m wind is then calculated as *U*_10_ = *U*_10*_ (*U*_10C_/*U*_10C*_), where *U*_10C_ is the neutral 10-m wind calculated from the COARE3.5 algorithm (*23*) and *U*_10C*_ is the COARE3.5 10-m wind without stability adjustment.

### Wave direction

The saildrone 20-Hz horizontal and 4-Hz vertical position data are used to calculate ocean surface wave direction in each 20-min segment, following (*49*)

$$\theta_{\text{wave}}={\text{tan}}^{-1}\frac{\text{corr}\left(v,\frac{d^{2}h}{dt^{2}}\right)}{\text{corr}\left(u,\frac{d^{2}h}{dt^{2}}\right)}$$

Here $\theta_{\text{wave}}$ is the wave direction from, clockwise from east, *v* is the vehicles’ northward velocity, *u* is eastward velocity, *h* is surface elevation, interpolated from 4 to 20 Hz, and corr is the Pearson correlation coefficient. Before calculating wave direction, the saildrone horizontal velocity and vertical acceleration are band-pass filtered to retain periods of 6 to 20 s, corresponding to the dominant swells. When comparing Wave Glider–derived bulk wave parameters to other in situ measurements in wave heights of 1 to 8 m, Thomson *et al.* (*49*) found ~5% errors and less than 5% bias. We found similarly small errors when comparing wave height and direction from a saildrone and a microSWIFT wave buoy (*29*) that were separated by about 30 km when they went through the core of Hurricane Milton west of Florida in October 2024 (fig. S12). Significant wave heights from the saildrone and buoy agree well, especially in the strongest winds and largest waves (fig. S12B). Peak wave heights from the buoy are larger, likely because the buoy was closer to the hurricane’s center. Wave direction from the saildrone also agrees well with the dominant wave direction from the buoy, especially in large waves (fig. S12C). In smaller seas, the agreement is worse, likely due to the combination of the weaker wave signal and differences between the saildrone and buoy locations (fig. S12A).

Because of the limited in situ wave data for direct comparison in winds (>15 m s^−1^), we also compare to output from WAVEWATCH III (*50*) forced with wind from either HWRF model (*51*) or HAFS-A (*52*). Outputs are hourly on a 0.1° grid from National Oceanic and Atmospheric Administration (NOAA)/NCEP/EMC (National Centers for Environmental Prediction/Environmental Modeling Center). We use forecast model runs initialized ~24 hours before peak wind speeds measured by the saildrones in hurricanes Sam (2021), Idalia (2023), Lee (2023), Ernesto (2024), Helene (2024), and Milton (2024). The model run for Sam was forced with HWRF, and the others were forced with HAFS-A. We use 20-min–averaged wave direction and winds from the saildrones. To minimize the impact of modeled track, intensity, and storm structure errors on the analysis, we first identify the center of the hurricane in the model based on the minimum wind speed within a radius of 60 km from the location of maximum wind speed. A transect in space is then taken through the modeled hurricane, with locations relative to storm center that correspond to the locations of the saildrone relative to the observed hurricane’s center, based on IBTrACS storm locations. Results show good agreement for wave direction, with discrepancies mostly when winds are weak (fig. S13).

To test the sensitivity of our *C*_d_ wind-wave results (Fig. 6) to wave frequency, we calculated wave direction using a range of different band-pass cutoff frequencies for vehicle horizontal motion and vertical acceleration. We then plotted the binned *C*_d_ versus *U*_10_ for aligned (<20° misalignment) and misaligned (>60°) cases. The results are shown in fig. S4B and indicate that our wave direction estimates are robust and that wind-wave misalignment consistently results in higher *C*_d_.

### Air bubbles

Seawater conductivity observations were obtained from a pumped Seabird Scientific SBE37-SMP-ODO Microcat sensor mounted on the saildrones’ keels at a depth of 1.6 m. The presence of air bubbles within the conductivity cell was inferred based on negative spikes in the 1-Hz conductivity. These spikes were identified using the criterion that the centered 3-s rolling SD exceeded 0.1 mS cm^−1^. Air bubbles were predominantly seen in TC transects and not observed outside of storms, consistent with air being a much poorer electrical conductor than seawater and bubbles mainly injected into the upper ocean during the stronger TCs. This is evident in the case of saildrone SD-1045’s 2021 mission shown below in fig. S13, where extremely low (7 to 50 mS/cm) conductivity measurements are seen only in the Hurricane Sam transect on 30 September, which was the only TC intercept during this particular mission. The extremely low spikes are confined to the storm intercept, although rain events were evident at other times in the mission, such as on 16 August, highlighted by a vehicle camera image in fig. S14A. This suggests that input of fresh water from rain cannot explain the low spikes in conductivity. The temporal distribution of bubble-affected (i.e., extremely low) and full-seawater (i.e., normal) conductivity measurements on 30 September (the storm-intercept day) is illustrated in more detail in fig. S14B, where the filtered (bubble) measurements are shown with larger, unfilled green circles and the seawater measurements are denoted with green circles that contain a blue dot.

The percentage of air bubble measurements within a given period (bubble occurrence %) was calculated by summing the number of 1-Hz air bubble measurements and dividing by the number of 1-Hz SD values contained in each minute of observation. Data from transects through all TCs listed in table S1 were used to generate the bubble occurrence results in Fig. 5, with the exception of Fiona (2022), for which conductivity was not available owing to sensor malfunction.

### Ocean surface brightness

We estimate ocean surface brightness using 97 saildrone images from seven different hurricanes. The calculation consists of first determining the location of the horizon in each image based on visual inspection. See fig. S8 for examples. With the horizon defined, we converted the red-green-blue images to hue-saturation-value and then calculated the mean “value” (i.e., brightness) over all ocean pixels. The calculation was limited to the middle 60% of pixels in the horizontal direction to eliminate any portion of the saildrone vehicle that might have been captured in the image.

### Error estimates

Uncertainties in *C*_d_ in each *U*_10_ bin are due primarily to the limited sample size of 20-min wind segments and the use of 20-min segments with up to 60% of the data missing. Therefore, the combined error is calculated as $E_{\text{tot}}=\sqrt{E_{s}^{2}+E_{m}^{2}}$. Here, *E_s_* is the SD of all *C*_d_ values in a given *U*_10_ bin and *E_m_* is the SD of ∆*C*_d_, which represents the difference between *C*_d_ calculated with 40 to 60% missing data and *C*_d_ calculated with <10% missing data (see fig. S11C). *E*_tot_ is shown as red shading in Fig. 5. For each *U*_10_ bin, we also calculate the SD of the 20-Hz *U*_10_ in each 20-min segment and use the mean SD as an estimate of the uncertainty in the binned *U*_10_. These uncertainties are shown as horizontal error bars in Fig. 5.

## Supplementary Materials

### This PDF file includes:

Figs. S1 to S14 Table S1 References
