# Supplementary material for: Hurricane air-sea drag saturation and sea-state dependence revealed by surface drones
Source: Sci Adv. 2026 May 27;12(22):eaec7422. doi: 10.1126/sciadv.aec7422 (PMC13215165; doi:10.1126/sciadv.aec7422)
Supplement: Supplementary file 1 — Figs. S1 to S14 Table S1 References [file sciadv.aec7422_sm.pdf]

Science Advances
AAAS

Supplementary Materials for

Hurricane air-sea drag saturation and sea-state dependence revealed by surface drones

Gregory R. Foltz *et al.*

Corresponding author: Gregory R. Foltz, gregory.foltz@noaa.gov

Sci. Adv. **12**, eaec7422 (2026)
DOI: 10.1126/sciadv.aec7422

This PDF file includes:

Figs. S1 to S14
Table S1
References

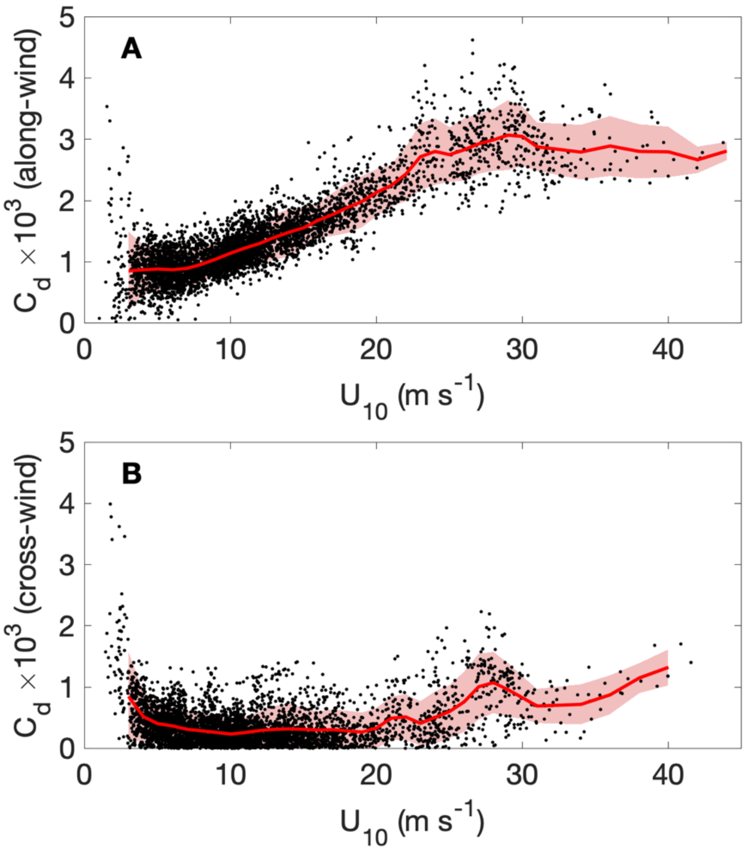

Fig. S1. Comparison of along-wind and cross-wind components of C_d. Black dots: C_d calculated in each 20-min segment using only the 20-Hz wind speed (**A**) along the direction of the mean wind and (**B**) across the direction of the mean wind. Red lines show averages in each 2 m s^{-1} bin with 1 m s^{-1} overlap for U_{10}<30 m s^{-1} and in 2 m s^{-1} bins with 2 m s^{-1} overlap for U_{10}>30 m s^{-1}. Red shading is one standard deviation.

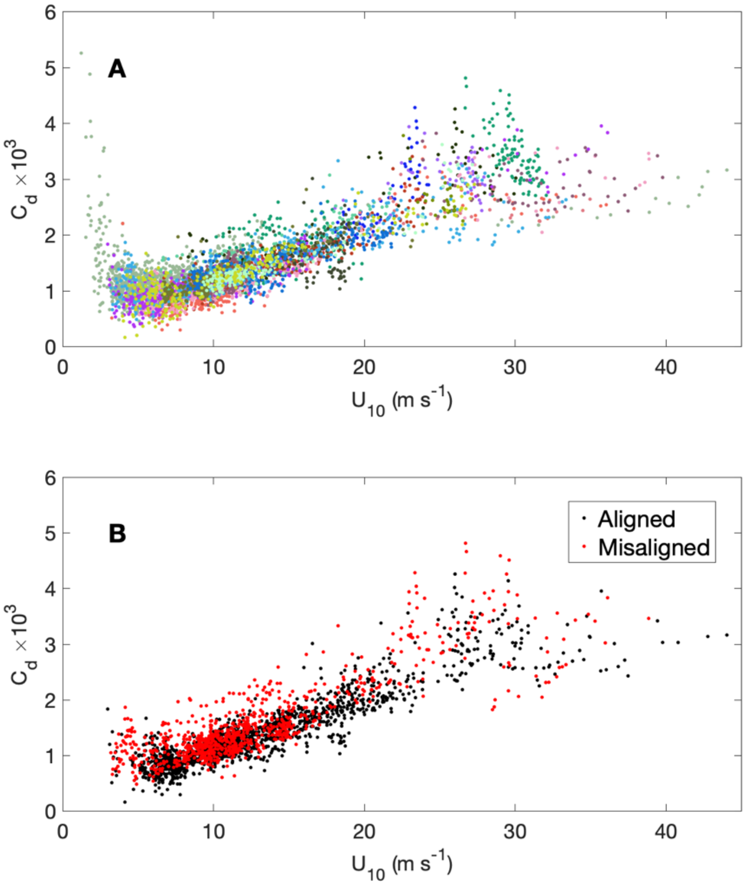

Fig. S2. C_d for individual hurricanes. (**A**) C_d calculated from each 20-min segment, with different colors indicating different hurricanes. (**B**) C_d from each 20-min segment with aligned (black dots, wind-wave angle <20°) and misaligned (red dots, >60°) wind and waves.

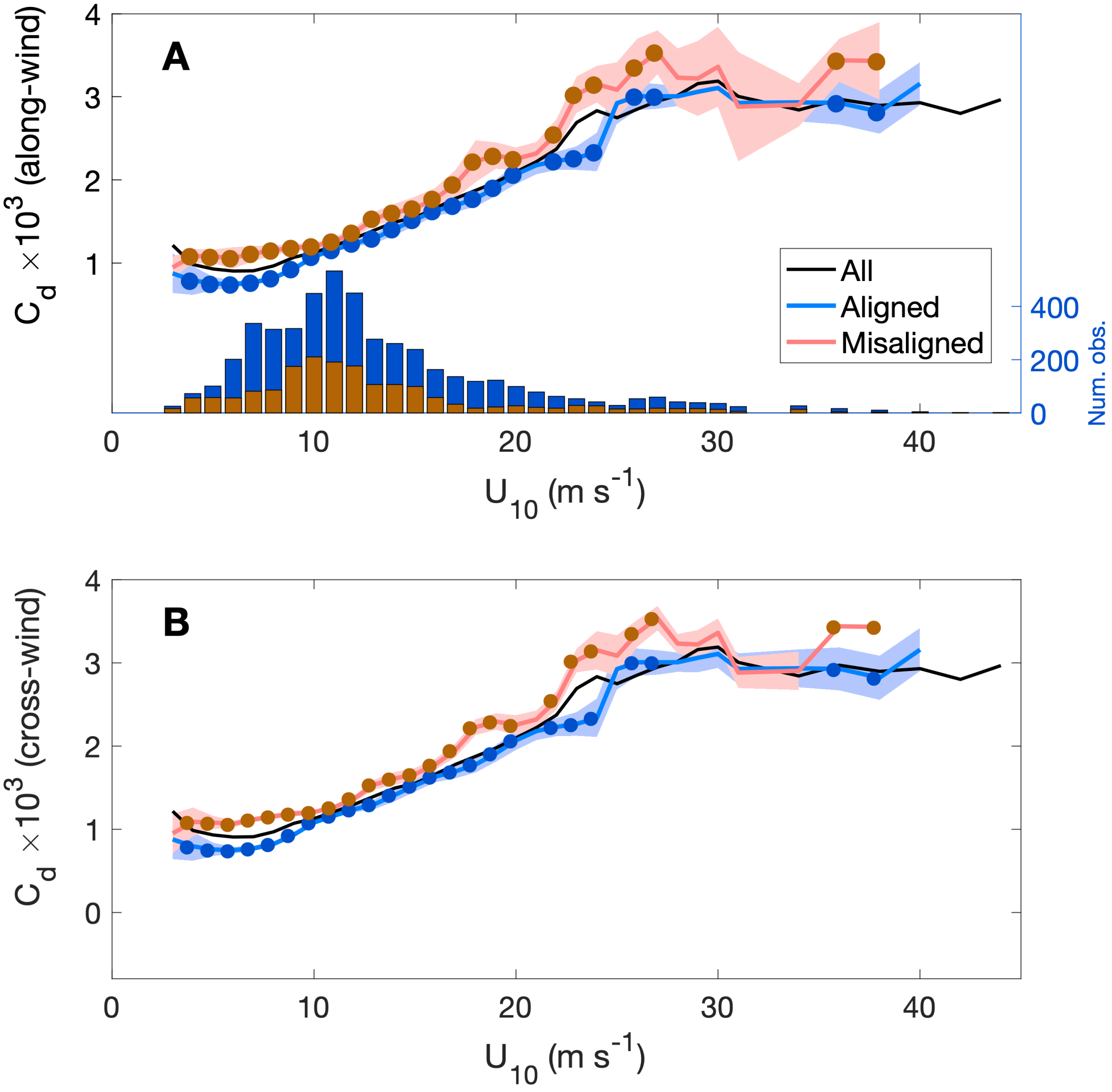

Fig. S3. Impact of wind-wave alignment on along- and cross-wind C_d. (**A**) Along-wind C_d for cases of aligned (wind-wave angle <20º) and misaligned (angle >60º) wind and waves (blue and red lines, respectively). Black line is C_d calculated from all data regardless of wind-wave angle. Dots indicate that a difference between aligned and misaligned C_d is significant at the 5% level. Blue and brown bars show the number of observations for aligned and misaligned cases, respectively. (**B**) Same as in (A) except for the cross-wind component of C_d. Numbers of observations are the same as in (A).

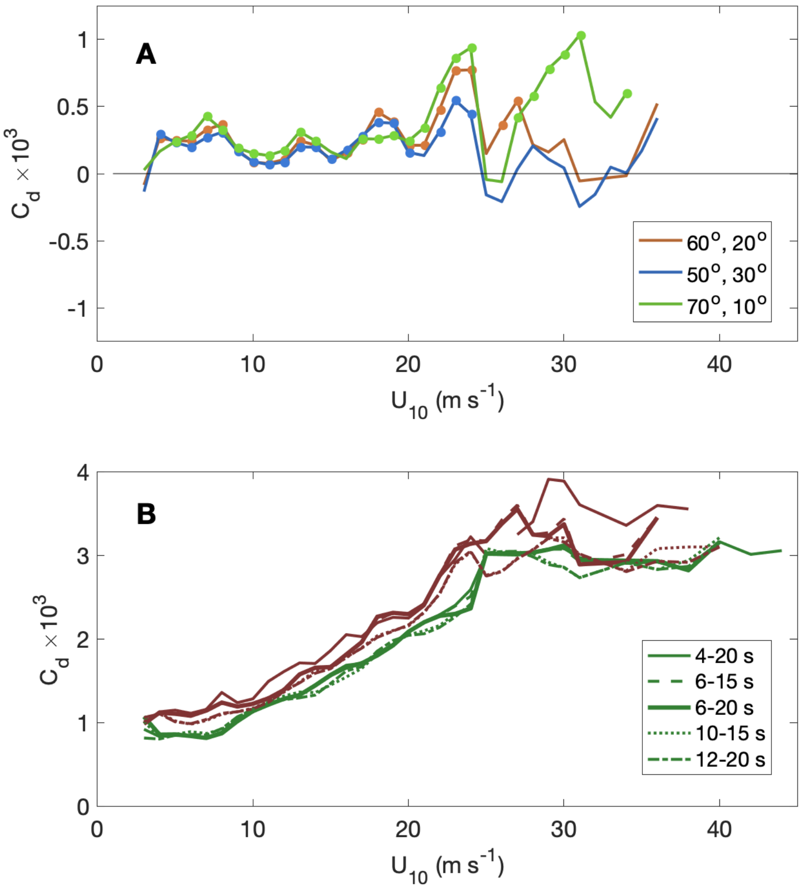

Fig. S4. Impact of wind-wave misalignment on C_d. (**A**) Differences in $\mathbf{C_d}$ between cases with misaligned and aligned wind and waves. Three different thresholds are used to define misaligned and aligned, respectively: >60° and <20° (red), >50° and <30° (blue), and >70° and <10° (green). Dots indicate that the difference in $\mathbf{C_d}$ between misaligned and aligned is significant at the 5% level. (**B**) Green lines: $\mathbf{C_d}$ averaged for aligned wind and waves using the different band-pass filter cutoff periods indicated in the legend. Red lines: same as green lines except averaged for misaligned cases.

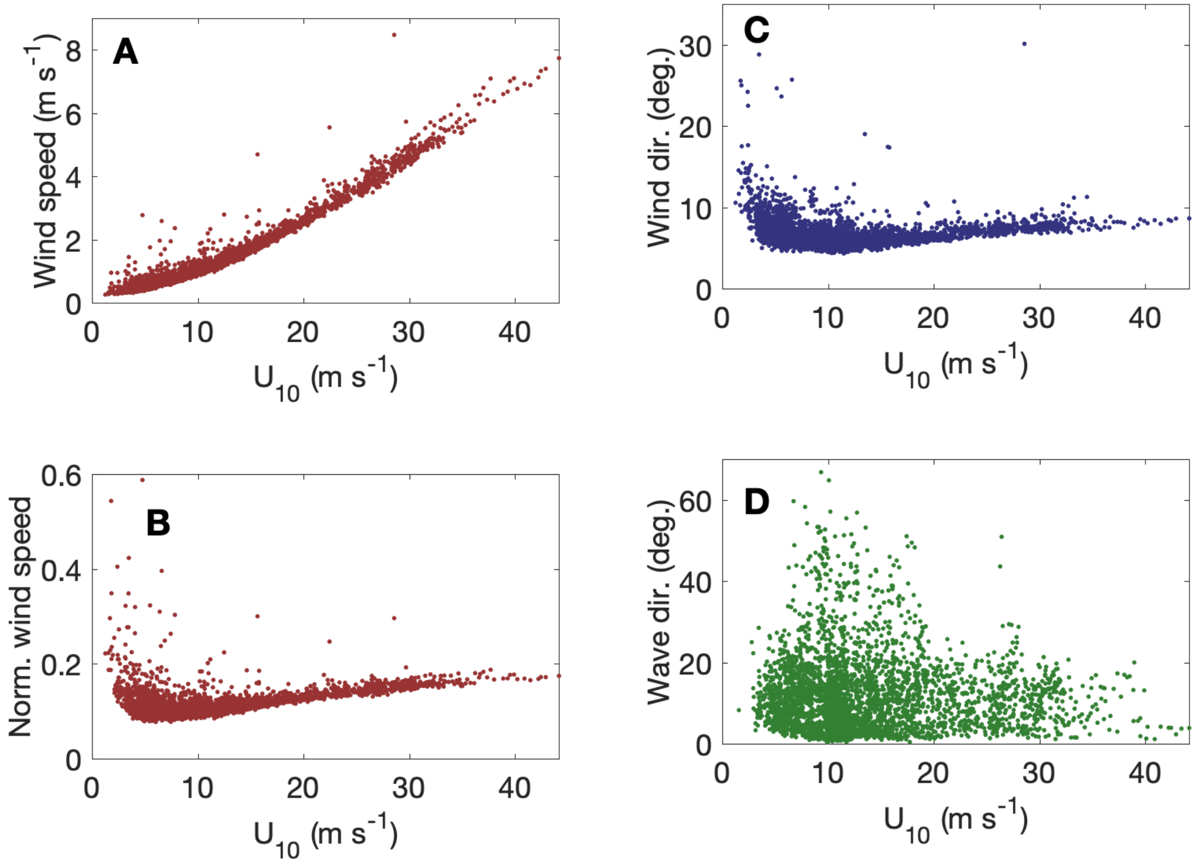

Fig. S5. Wind and wave variability. (**A**) Standard deviation of wind speed. (**B**) Standard deviation of wind speed normalized by the mean U_{10}. Standard deviations of (**C**) wind direction and (**D**) wave direction. Standard deviations in (A)-(C) were calculated using all 20-Hz wind data in each 20-min segment. In (D), standard deviations were calculated using all 2-min periods within each 20-min segment.

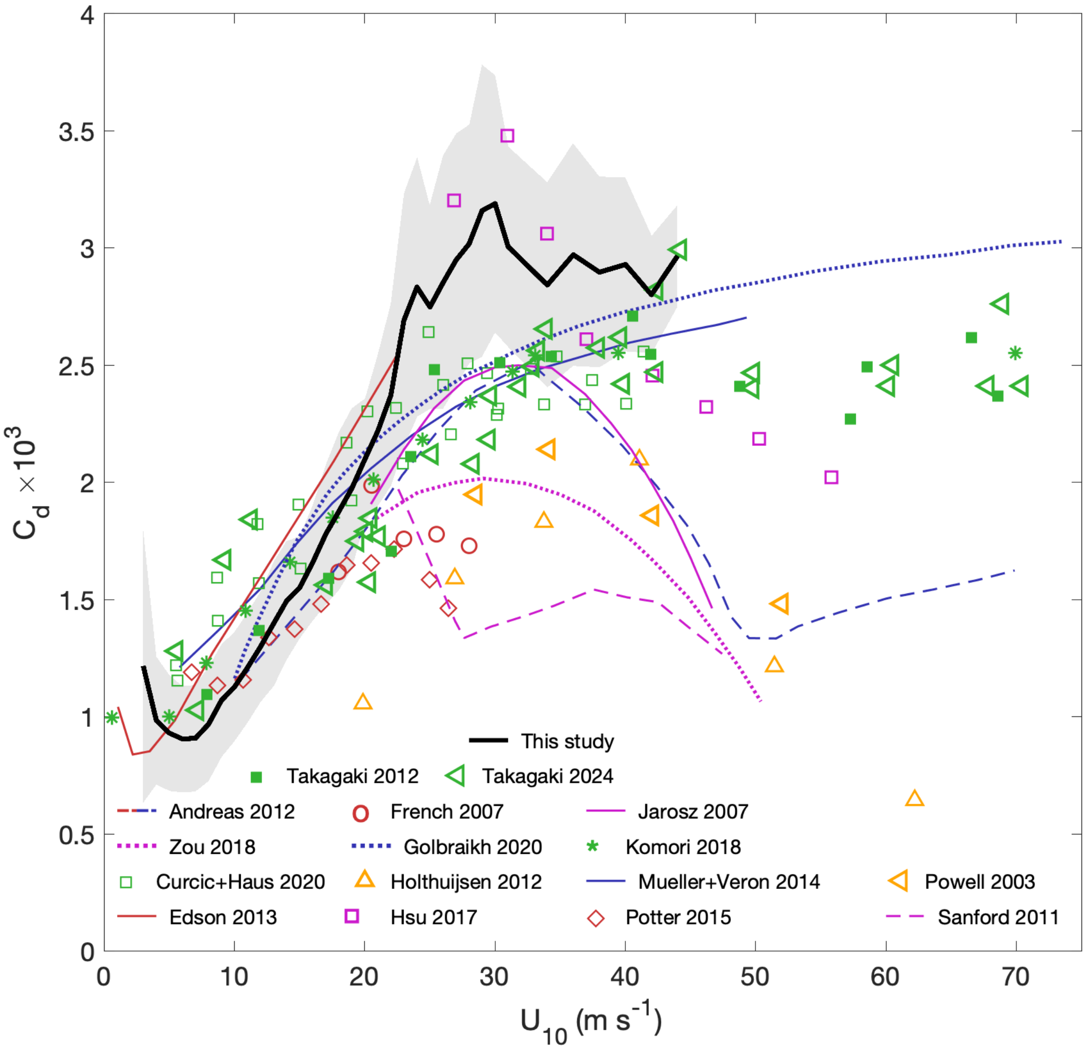

Fig. S6. C_d results from previous studies compared this study. Lines and symbols are colored based on the technique used to estimate C_d: Red for direct covariance, orange for vertical wind profile, green for wave tank, blue for model/theory, and purple for ocean momentum balance. Gray shading is one standard deviation from the mean for the results from this study (black curve). To save space in the legend, "et al." was omitted from studies with three or more authors. None of the studies has only one author. Andreas et al. 2012, Takagaki et al. 2012, Takagaki et al. 2024, Golbraikh et al. 2020, Zou et al. 2018, Komori et al. 2018, Mueller and Veron 2014, and Sanford et al. 2011 correspond to (*54 - 61*), respectively.

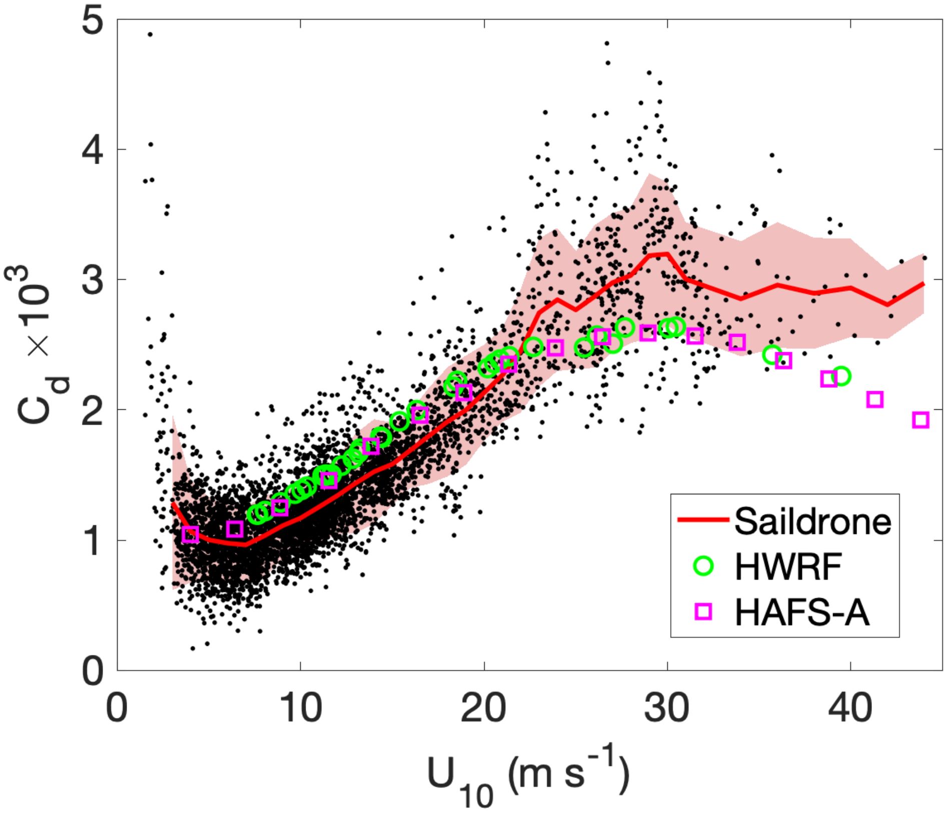

Fig. S7. C_d used in forecast models. Comparison of $\mathbf{C_d}$ from the present study (black dots and red line with shading) to the $\mathbf{C_d}$ used in the HWRF (green circles) and HAFS-A (magenta circles) operational forecast models.

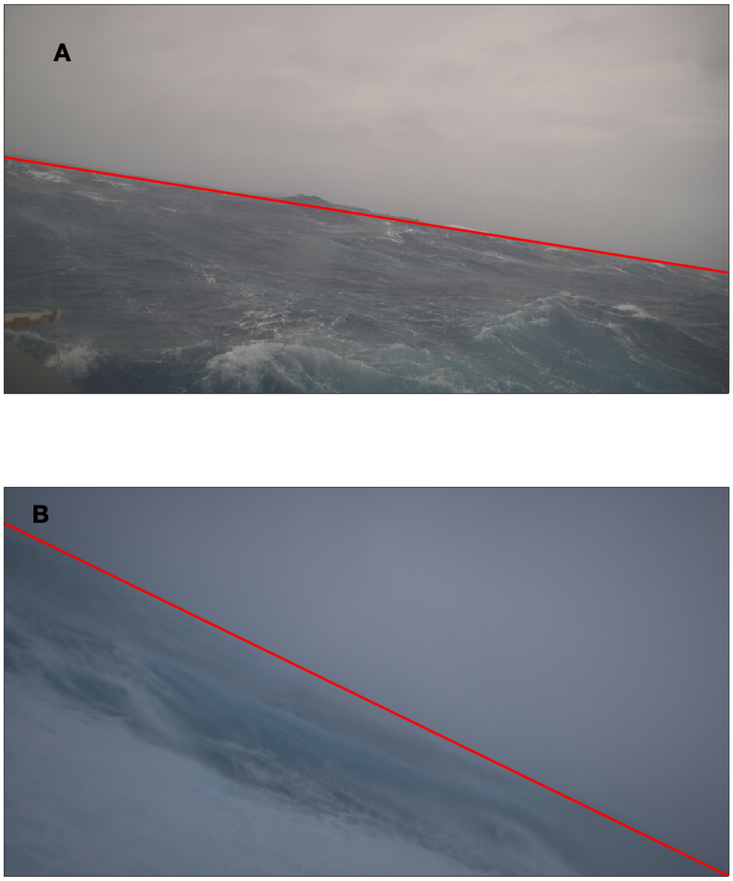

Fig. S8. Air-sea horizon definition from images taken by Saildrone Explorer uncrewed surface vehicles. Examples of visual determination of the horizon (red lines) for cases with (**A**) U_{10} = 21 m s^{-1} and (**B**) U_{10} = 39 m s^{-1}.

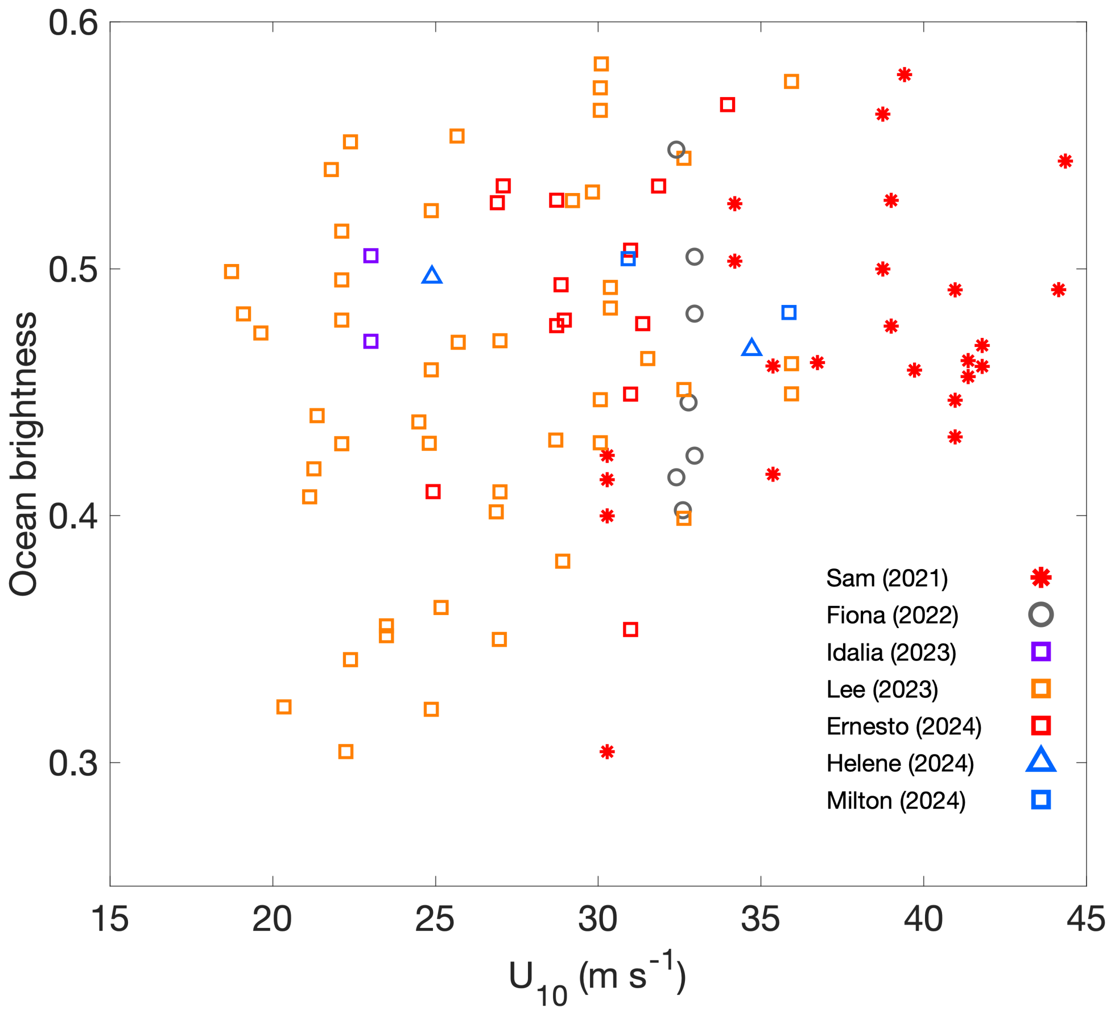

Fig. S9. Changes in ocean surface brightness with wind speed. Values are color-coded based on the hurricane from which the data were acquired. Brightness was calculated for each pixel of each saildrone image and then averaged only over the ocean pixels (see Materials and Methods and Fig. S8).

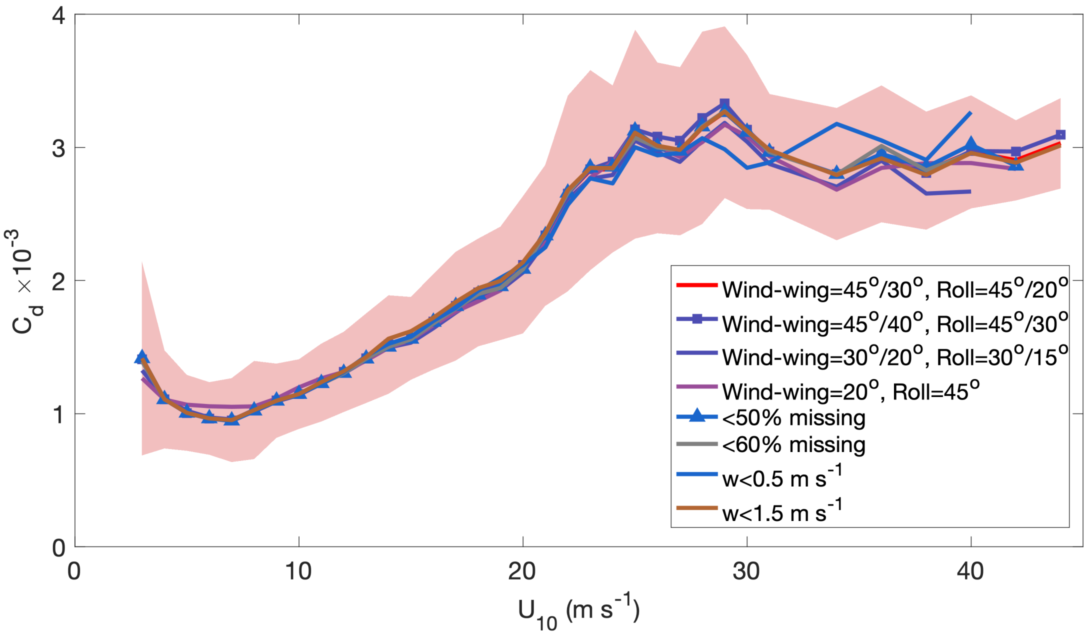

Fig. S10. Sensitivity of C_d to different QC thresholds. The first three curves show results for varying wind-wing angle thresholds, labeled as "instantaneous 20-Hz value / 1-min. mean absolute value." For "Wind-wing=20°, Roll=45°," both numbers refer to 20-Hz thresholds. Also shown are results for the default thresholds except requiring <50% of the 20-Hz data missing in each 20-min. segment and <60% missing, instead of the default of 40%, and requiring mean 1- min. vertical wind <0.5 m s^{-1} and <1.5 m s^{-1} instead of the default of <1 m s^{-1}. Red shading is one standard deviation from the mean using the default thresholds.

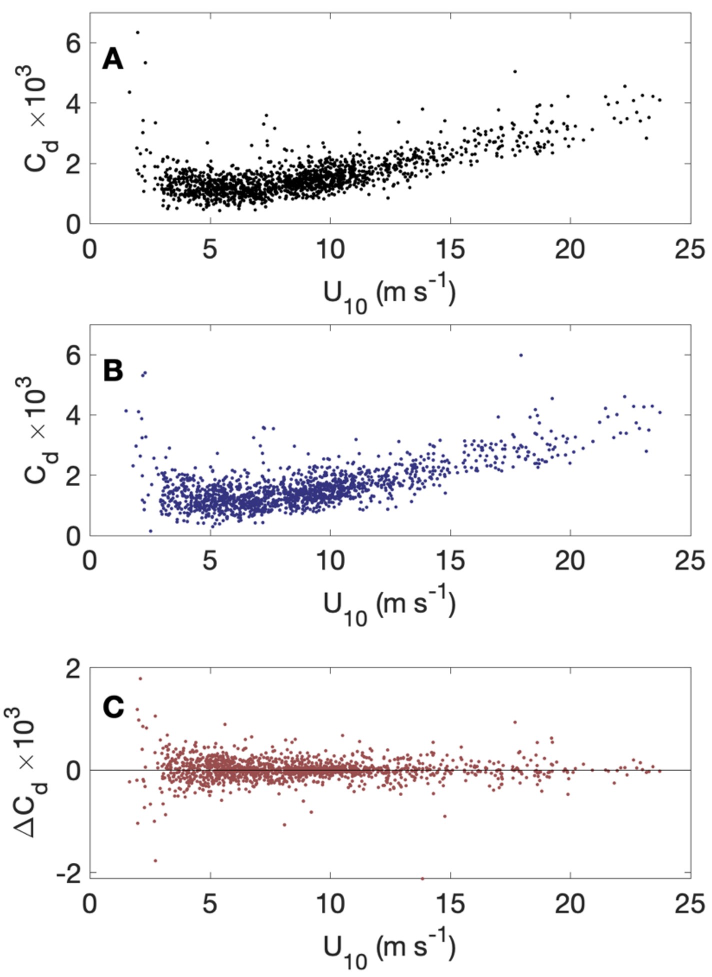

Fig. S11. Sensitivity of C_d to missing data. (**A**) C_d calculated in each 20-min segment with >90% of the 20-Hz data available. (**B**) C_d calculated for the same segments but with 40-60% of the 20-Hz data removed. (**C**) Difference between C_d in (A) and (B).

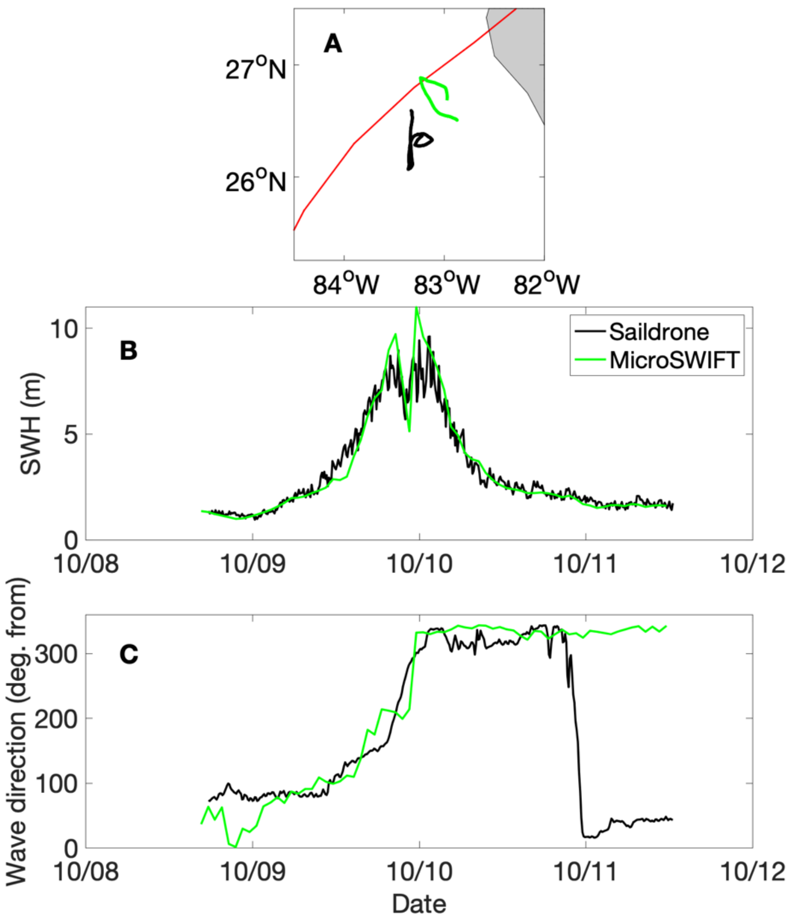

Fig. S12. Wave direction buoy comparison. (**A**) Paths of a saildrone (black) and drifting buoy (green) during the time period shown in (B) and (C). Red line shows the path of Hurricane Milton. (**B**) Significant wave height measured by the saildrone (black) and buoy (green) during October 2024. (**C**) Same as (B) except wave direction. In (B) and (C), the saildrone time series have been shifted one hour later to match the time of the buoy's intercept of Hurricane Milton.

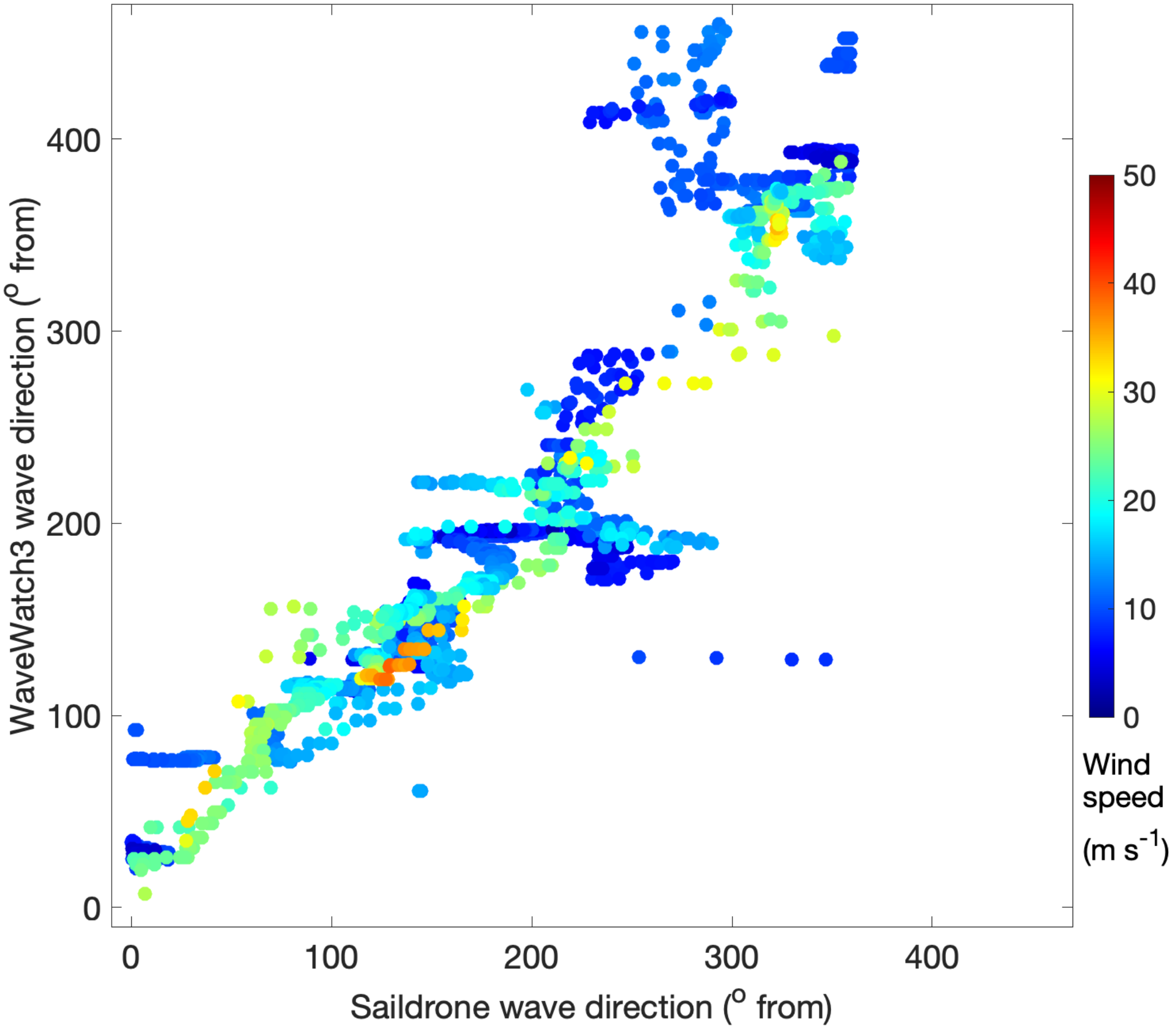

Fig. S13. Comparison of wave direction from saildrones and WaveWatch III (WW3).
Primary wave direction from WW3 (y axis) compared to swell direction from saildrones (x axis), measured clockwise from north. Colors indicate wind speed measured by the saildrone and then adjusted to a height of 10 m.

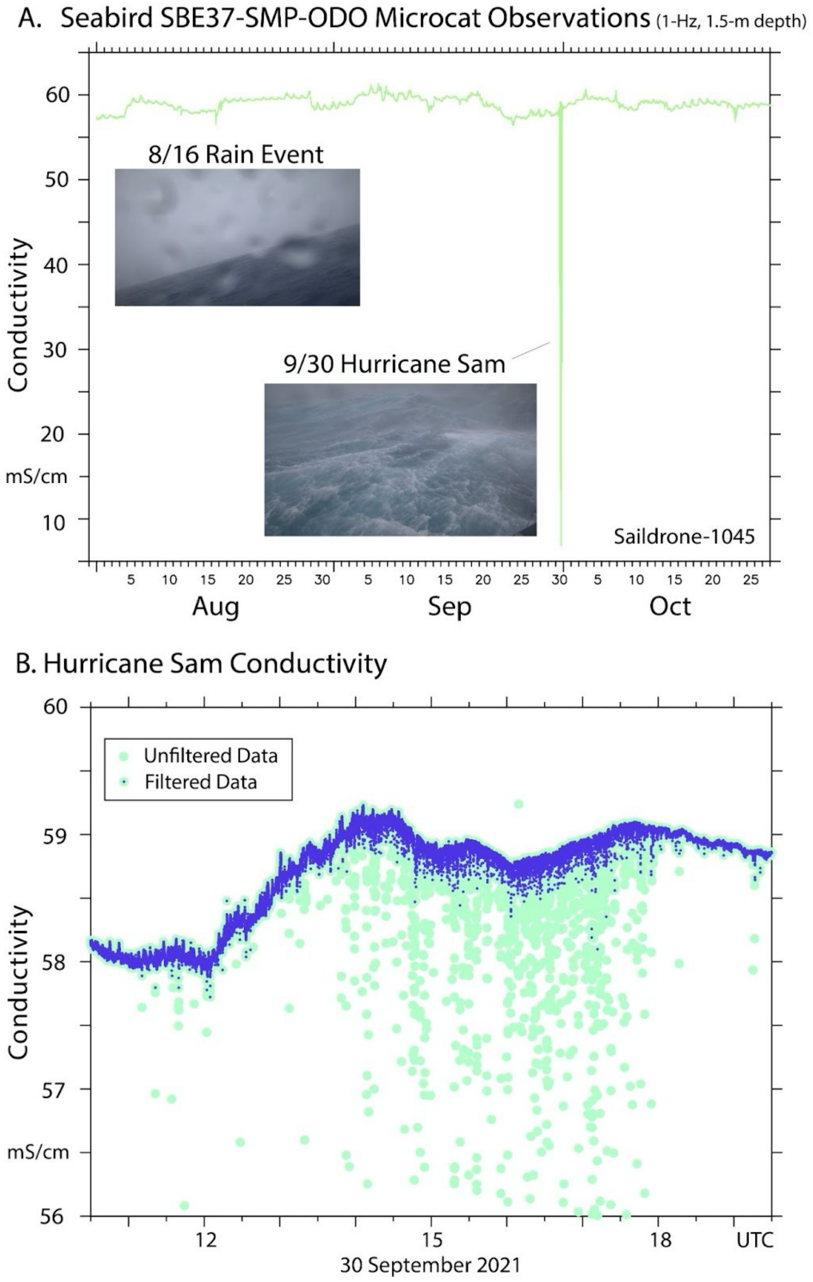

Fig. S14. Example of air bubble estimation. (A) The 7,602,200 1-Hz observations collected by Saildrone-1045 during the 2021 Hurricane Monitoring Mission, with a rain event on 8/16 and the intercept of Hurricane Sam on 9/30 highlighted with Saildrone Explorer uncrewed surface vehicle images. **(B)** 1-Hz conductivity observations during 9/30. The green circles with smaller dark-blue circles embedded show the observations that remain after applying a 3-second rolling window standard deviation filter, with a threshold of 0.1 mS/cm. Here, these are taken as the measurements indicative of actual seawater salinity, and the filtered-out values indicative of measurements taken with air bubbles in the conductivity cell.

Tropical Cyclone	Max wind ($m\ s^{-1}$)	Max SWH (m)	Minutes 18 $m\ s^{-1}$	Minutes 26 $m\ s^{-1}$	Minutes 33 $m\ s^{-1}$
Sam (2021) SD-1045	41 (48)	14.2	360 (498)	167 (205)	100 (143)
Fiona (2022) SD-1078	35 (41)	15.4	330 (339)	110 (203)	7 (55)
Ian (2022) SD-1059	29 (34)	8.6	322 (366)	21 (168)	0 (3)
Idalia (2023) SD-1045	23 (27)	6.2	71 (261)	0 (0)	0 (0)
Idalia (2023) SD-1057	24 (28)	7.9	247 (502)	0 (7)	0 (0)
Idalia (2023) SD-1083	36 (42)	9.6	216 (265)	67 (118)	1 (37)
Lee (2023) SD-1036	31 (36)	11.4	1012 (1216)	310 (937)	1 (58)
Ophelia (2023) SD-1057	21 (24)	6.3	20 (477)	0 (0)	0 (0)
Ophelia (2023) SD-1045	22 (26)	8.5	297 (460)	0 (2)	0 (0)
Tammy (2023) SD-1041	28 (33)	6.9	472 (535)	7 (230)	0 (0)
Ernesto (2024) SD-1069	24 (28)	8.9	243 (498)	0 (0)	0 (0)
Ernesto (2024) SD-1091	27 (32)	6.7	178 (237)	3 (51)	0 (0)
Ernesto (2024) SD-1068	31 (36)	10.9	663 (888)	65 (314)	0 (26)
Helene (2024) SD-1083	33 (39)	9.5	162 (165)	87 (156)	0 (52)
Helene (2024) SD-1057	35 (41)	7.3	252 (327)	104 (212)	24 (72)
Milton (2024) SD-1042	26 (31)	9.1	238 (305)	1 (88)	0 (0)

Milton (2024) SD-1057	28 (33)	8.3	56 (130)	0 (2)	0 (0)
Milton (2024) SD-1083	38 (45)	10.1	564 (570)	311 (487)	33 (202)
Rafael (2024) SD-1057	26 (31)	8.3	243 (251)	1 (78)	0 (0)

Table S1: Wind and wave measurement summaries from saildrone TC intercepts. Column 1: Tropical cyclone name and saildrone vehicle number. Columns 2: Maximum 1-minute averaged measured wind speed, with maximum measured wind speed adjusted to 10 m height in parentheses. Column 3: 15-minute significant wave height (SWH). Columns 4-6: Number of minutes with mean measured wind speed exceeding tropical storm force (18 m s^{-1}), 50 kt (26 m s^{-1}), and hurricane force (33 m s^{-1}), after final quality control. Numbers in parentheses are for wind speed adjusted to 10 m height.

REFERENCES

1. K. A. Emanuel, An air-sea interaction theory for tropical cyclones. Part I: Steady-state maintenance. *J. Atmos. Sci.* **43**, 585–605 (1986).

2. J. J. Cione, E. W. Uhlhorn, Sea surface temperature variability in hurricanes: Implications with respect to intensity change. *Mon. Weather Rev.* **131**, 1783–1796 (2003).

3. I.-J. Moon, I. Ginis, T. Hara, B. Thomas, A physics-based parameterization of air–sea momentum flux at high wind speeds and its impact on hurricane intensity predictions. *Mon. Weather Rev. 135*, 2869–2878 (2007).

4. W. M. Drennan, H. C. Graber, D. Hauser, C. Quentin, On the wave age dependence of wind stress over pure wind seas. *J. Geophys. Res.* **108**, 8062 (2003).

5. L. H. Holthuijsen, M. D. Powell, J. D. Pietrzak, Wind and waves in extreme hurricanes. *J. Geophys. Res.* **117**, C09003 (2012).

6. S. S. Chen, W. Zhao, M. A. Donelan, H. L. Tolman, Directional wind–wave coupling in fully coupled atmosphere–wave–ocean models: Results from CBLAST-Hurricane. *J. Atmos. Sci.* **70**, 3198–3215 (2013).

7. B. G. Reichl, T. Hara, I. Ginis, Sea state dependence of the wind stress over the ocean under hurricane winds. *J. Geophys. Res.* **119**, 30–51 (2014).

8. M. D. Powell, P. J. Vickery, T. A. Reinhold, Reduced drag coefficient for high wind speeds in tropical cyclones. *Nature 422*, 279–283 (2003).

9. E. D. Jarosz, A. Mitchell, D. W. Wang, W. J. Teague, Bottom-up determination of air-sea momentum exchange under a major tropical cyclone. *Science* **315**, 1707–1709 (2007).

10. J.-Y. Hsu, R.-C. Lien, E. A. D'Asaro, T. B. Sanford, Estimates of surface wind stress and drag coefficients in Typhoon Megi. *J. Phys. Oceanogr.* **47**, 545–565 (2017).

11. M. A. Donelan, B. K. Haus, N. Reul, W. J. Plant, M. Stiassnie, H. C. Graber, O. B. Brown, E. S. Saltzman, On the limiting aerodynamic roughness of the ocean in very strong winds. *Geophys. Res. Lett.* **31**, L18306 (2004).

12. M. Curcic, B. K. Haus, Revised estimates of ocean surface drag in strong winds. *Geophys. Res. Lett.* **47**, e2020GL087647 (2020).

13. J. R. French, W. M. Drennan, J. A. Zhang, P. G. Black, Turbulent fluxes in the hurricane boundary layer. Part I: Momentum flux. *J. Atmos. Sci.* **64**, 1089–1102 (2007).

14. H. Potter, H. C. Graber, N. J. Williams, C. O. Collins III, R. J. Ramos, W. M. Drennan, In situ measurements of momentum fluxes in typhoons. *J. Atmos. Sci.* **72**, 104–118 (2015).

15. D. Zhang, A. Chiodi, C. Zhang, G. R. Foltz, M. Cronin, C. W. Mordy, J. Cross, E. Cokelet, J. Zhang, C. Meinig, N. Lawrence-Slavas, P. Stabeno, R. Jenkins, Observing extreme ocean and weather events using innovative saildrone uncrewed surface vehicles. *Oceanography* **36**, 70–77 (2023).

16. C. Zhang, G. R. Foltz, A. Chiodi, C. Mordy, C. Edwards, C. Meinig, D. Zhang, E. Mazza, E. Cokelet, E. Burger, F. Bringas, G. Goni, H. Hristova, H.-S. Kim, J. Trinanes, J. A. Zhang, K. Bailey, K. O'Brien, M. Morales-Caez, N. Lawrence-Slavas, R. Jenkins, S. Chen, X. Chen, Hurricane observations by uncrewed systems. *Bull. Am. Meteorol. Soc.* **104**, E1893–E1917 (2023).

17. M. C. Schönau, T. Paluszkiewicz, L. R. Centurioni, W. A. Komaromi, H. Jin, J. D. Doyle, In situ observations at the air-sea interface by expendable air-deployed drifters under Hurricane Michael (2018). *Geophys. Res. Lett.* **51**, e2023GL105730 (2024).

18. M. Miyake, R. W. Stewart, R. W. Burling, Spectra and cospectra of turbulence over water. *Q. J. Roy. Meteorol. Soc.* **96**, 138–143 (1970).

19. J. A. Zhang, Spectral characteristics of turbulence in the hurricane boundary layer over the ocean between the outer rain bands. *Q. J. R. Meteorol. Soc.* **136**, 918–926 (2010).

20. P. S. DeLeonibus, Momentum flux and wave spectra observations from an ocean tower. *J. Geophys. Res.* **76**, 6506–6527 (1971).

21. R. A. Antonia, A. J. Chambers, Wind-wave-induced disturbances in the marine surface layer. *J. Phys. Oceanogr.* **10**, 611–622 (1980).

22. L. Grare, L. Lenain, W. K. Melville, Vertical profiles of the wave-induced airflow above ocean surface waves. *J. Phys. Oceanogr.* **48**, 2901–2922 (2018).

23. J. B. Edson, V. Jampana, R. A. Weller, S. P. Bigorre, A. J. Plueddemann, C. W. Fairall, S. D. Miller, L. Mahrt, D. Vickers, H. Hersbach, On the exchange of momentum over the open ocean. *J. Phys. Oceanogr.* **43**, 1589–1610 (2013).

24. P. A. Hwang, Y.-H. L. Hsu, J. Wu, Air bubbles produced by breaking wind waves: A laboratory study. *J. Phys. Oceanogr.* **20**, 19–28 (1990).

25. J.-H. Liang, J. C. McWilliams, P. P. Sullivan, B. Baschek, Large eddy simulation of the bubbly ocean: New insights on subsurface bubble distribution and bubble-mediated gas transfer. *J. Geophys. Res.* **117**, C04002 (2012).

26. Y. Troitskaya, D. Sergeev, A. Kandaurov, M. Vdovin, S. Zilitinkevich, The effect of foam on waves and the aerodynamic roughness of the water surface at high winds. *J. Phys. Oceanogr.* **49**, 959–981 (2019).

27. E. L. Andreas, K. A. Emanuel, Effects of sea spray on tropical cyclone intensity. *J. Atmos. Sci.* **58**, 3741–3751 (2001).

28. P. G. Black, E. A. D'Asaro, W. M. Drennan, J. R. French, P. P. Niiler, T. B. Sanford, E. J. Terrill, E. J. Walsh, J. A. Zhang, Air-sea exchange in hurricanes: Synthesis of observations from the Coupled Boundary Layer Air-Sea Transfer Experiment. *Bull. Am. Meteorol. Soc.* **88**, 357–374 (2007).

29. J. R. Davis, J. Thomson, I. A. Houghton, C. W. Fairall, B. J. Butterworth, E. J. Thompson, G. de Boer, J. D. Doyle, J. R. Moskaitis, Ocean surface wave slopes and wind-wave alignment observed in Hurricane Idalia. *J. Geophys. Res. Oceans* **130**, e2024JC021814 (2025).

30. C. Sauvage, H. Seo, B. W. Barr, J. B. Edson, C. A. Clayson, Misaligned wind-waves behind atmospheric cold fronts. *J. Geophys. Res. Oceans* **129**, e2024JC021162 (2024).

31. A. A. Grachev, C. W. Fairall, J. E. Hare, J. B. Edson, S. D. Miller, Wind stress vector over ocean waves. *J. Phys. Oceanogr.* **33**, 2408–2429 (2003).

32. S. Porchetta, O. Temel, D. Muñoz-Esparza, J. Reuder, J. Monbaliu, J. van Beeck, N. van Lipzig, A new roughness length parameterization accounting for wind–wave (mis)alignment. *Atmos. Chem. Phys.* **19**, 6681–6700 (2019).

33. M. K. Biswas, S. Abarca, L. Bernardet, I. Ginis, E. Grell, M. Iacono, E. Kalina, B. Liu, Q. Liu, T. Marchok, A. Mehra, K. Newman, J. Sippel, V. Tallapragada, B. Thomas, W. Wang, H. Winterbottom, Z. Zhang, "Hurricane Weather Research and Forecasting (HWRF) model: 2018 scientific documentation" (DTC, 2018); https://dtcenter.org/sites/default/files/community-code/hwrf/docs/scientific_documents/HWRFv4.0a_ScientificDoc.pdf.

34. W. Wang, J. Han, J. Shin, X. Chen, A. Hazelton, L. Zhu, H.-S. Kim, X. Li, B. Liu, Q. Liu, J. Steffen, R. Sun, W. Zheng, Z. Zhang, F. Yang, Physics schemes in the first version of NCEP operational hurricane analysis and forecast system (HAFS). *Front. Earth Sci.* **12**, 1379069 (2024).

35. D. H. Richter, C. Wainwright, D. P. Stern, G. H. Bryan, D. Chavas, Potential low bias in high-wind drag coefficient inferred from dropsonde data in hurricanes. *J. Atmos. Sci.* **78**, 2339–2352 (2021).

36. D. G. Ortiz-Suslow, J. Kalogiros, R. Yamaguchi, Q. Wang, An evaluation of the constant flux layer in the atmospheric flow above the wavy air-sea interface. *J. Geophys. Res.* **126**, e2020JD032834 (2021).

37. L. Grare, L. Lenain, J. T. Farrar, Observing ocean-atmosphere fluxes from autonomous surface vehicles. *Geophys. Res. Lett.* **52**, e2025GL115335 (2025).

38. I. B. Savelyev, "A laboratory study of the transfer of momentum across the air-sea interface in strong winds," thesis, University of Miami, Miami, FL (2009).

39. T. Hara, P. P. Sullivan, Wave boundary layer turbulence over surface waves in a strongly forced condition. *J. Phys. Oceanogr.* **45**, 868–883 (2015).

40. J. B. Edson, A. A. Hinton, K. E. Prada, J. E. Hare, C. W. Fairall, Direct covariance flux estimates from mobile platforms at sea. *J. Atmos. Ocean. Technol.* **15**, 547–562 (1998).

41. D. Zhang, M. F. Cronin, C. Meinig, J. T. Farrar, R. Jenkins, D. Peacock, J. Keene, A. Sutton, Q. Yang, Comparing air-sea flux measurements from a new unmanned surface vehicle and proven platforms during the SPURS-2 field campaign. *Oceanography* **32**, 122–133 (2019).

42. J. E. Reeves Eyre, M. F. Cronin, D. Zhang, E. J. Thompson, C. W. Fairall, J. B. Edson, Saildrone direct covariance wind stress in various wind and current regimes of the tropical Pacific. *J. Atmos. Ocean. Technol.* **40**, 503–517 (2023).

43. R. G. Patterson, M. F. Cronin, S. Swart, J. Beja, J. M. Edholm, J. McKenna, J. B. Palter, A. Parker, C. I. Addey, W. Boone, P. Bhuyan, J. J. H. Buck, E. F. Burger, J. Burris, L. Camus, B. de Young, M. du Plessis, M. Flanigan, G. R. Foltz, S. T. Gille, L. Grare, J. E. Hansen, L. R. Hole, M. C. Honda, V. Hormann, C. Kohlman, N. Kosaka, C. Kuhn, L. Lenain, L. Looney, A. Marouchos, E. K. McGeorge, C. R. McMahon, S. Mitarai, C. Mordy, A. Nagano, S.-A. Nicholson, S. Nickford, K. M. O'Brien, D. Peddie, L. Ponsoni, V. Ramasco, N. Rozenauers, E. Siddle, C. Stienbarger, A. J. Sutton, N. Tada, J. Thomson, I. Ueki, L. Yu, C. Zhang, D. Zhang, Uncrewed surface vehicles in the Global Ocean Observing System: A new frontier for observing and monitoring at the air-sea interface. *Front. Mar. Sci.* **12**, 1523585 (2025).

44. G. R. Foltz, C. Zhang, C. Meinig, J. A. Zhang, D. Zhang, An unprecedented view inside a hurricane. *Eos* **103**, 22–28 (2022).

45. K. R. Knapp, M. C. Kruk, D. H. Levinson, H. J. Diamond, C. J. Neumann, The International Best Track Archive for Climate Stewardship (IBTrACS): Unifying tropical cyclone best track data. *Bull. Am. Meteorol. Soc.* **91**, 363–376 (2010).

46. J. Gahtan, K. R. Knapp, C. J. Schreck, H. J. Diamond, J. P. Kossin, M. C. Kruk, International Best Track Archive for Climate Stewardship (IBTrACS) project, version 4r01, North Atlantic

subset, NOAA National Centers for Environmental Information (2024); doi:10.25921/82ty-9e16 [accessed 21 Nov 2024].

47. A. M. Chiodi, H. Hristova, G. R. Foltz, J. A. Zhang, C. W. Mordy, C. R. Edwards, C. Zhang, C. Meinig, D. Zhang, E. Mazza, E. D. Cokelet, E. F. Burger, F. Bringas, G. Goni, H.-S. Kim, J. Trinanes, K. Bailey, K. M. O'Brien, M. Morales-Caez, N. Lawrence-Slavas, S. S. Chen, X. Chen, Surface ocean warming near the core of Hurricane Sam and its representation in forecast models. *Front. Mar. Sci.* **10**, 1297974 (2024).

48. Y. Chang, A. Kirincich, J. B. Edson, Observations of coastal wind momentum flux: Dependence on fetch and waves with comparisons to COARE. *J. Phys. Oceanogr.* **55**, 1321–1334 (2025).

49. J. Thomson, G. B. Girton, R. Jha, A. Trapani, Measurements of directional wave spectra and wind stress from a Wave Glider autonomous surface vehicle. *J. Atmos. Ocean. Technol.* **35**, 347–363 (2018).

50. H. L. Tolman, "User manual and system documentation of WAVEWATCH III version 4.18," (NOAA/NWS/NCEP/MMAB Tech. Note 316, NOAA, 2014).

51. S. G. Gopalakrishnan, Q. Liu, T. Marchok, D. Sheinin, N. Surgi, R. Tuleya, R. Yablonsky, X. Zhang, "Hurricane Weather Research and Forecasting (HWRF) Model: 2011 scientific documentation," (NOAA/NCAR/Development Testbed Center, 2011).

52. A. Hazelton, G. J. Alaka Jr., L. Gramer, W. Ramstrom, S. Ditchek, X. Chen, B. Liu, Z. Zhang, L. Zhu, W. Wang, B. Thomas, J. Shin, C.-K. Wang, H.-S. Kim, X. Zhang, A. Mehra, F. Marks, *2022 real-time hurricane forecasts from an experimental version of the Hurricane Analysis and Forecast System (HAFSV0.3S)*, *Front. Earth Sci.* **11**, 1264969 (2023).

53. G. R. Foltz, Data and scripts to accompany: Hurricane air-sea drag saturation and sea-state dependence revealed by surface drones, Zenodo (2026); 10.5281/zenodo.19632118.

54. E. L. Andreas, L. Mahrt, D. Vickers, A new drag relation for aerodynamically rough flow over the ocean. *J. Atmos. Sci.* **69**, 2520–2537 (2012).

55. N. Takagaki, S. Komori, N. Suzuki, K. Iwano, T. Kuramoto, S. Shimada, R. Kurose, K. Takahashi, Strong correlation between the drag coefficient and the shape of the wind sea spectrum over a broad range of wind speeds. *Geophys. Res. Lett.* **39**, L23604 (2012).

56. N. Takagaki, N. Suzuki, K. Iwano, K. Nishiumi, R. Hayashi, N. Kurihara, K. Nishitani, T. Hamaguchi, Fetch effects on air-sea momentum transfer at very high wind speeds. *Coast. Eng. J.* **66**, 139–152 (2024).

57. E. Golbraikh, Y. M. Shtemler, Momentum and heat transfer across the foam-covered air-sea interface in hurricanes. *Ocean Dyn. 70*, 683–692 (2020).

58. Z. Zou, D. Zhao, J. Tian, B. Liu, J. Huang, Drag coefficients derived from ocean current and temperature profiles at high wind speeds. *Tellus* **70**, 1–13 (2022).

59. S. Komori, K. Iwano, N. Takagaki, R. Onishi, R. Kurose, K. Takahashi, N. Suzuki, Laboratory measurements of heat transfer and drag coefficients at extremely high wind speeds. *J. Phys. Oceanogr.* **48**, 959–974 (2018).

60. J. A. Mueller, F. Veron, Impact of sea spray on air–sea fluxes. Part II: Feedback effects. *J. Phys. Oceanogr. 44*, 2835–2853 (2014).

61. T. B. Sanford, J. F. Price, J. B. Girton, Upper-ocean response to Hurricane Frances (2004) observed by profiling EM-APEX floats. *J. Phys. Oceanogr.* **41**, 1041–1056 (2011).
